# Suppressor of Cytokine Signalling-6 Promotes Neurite Outgrowth via JAK2/STAT5-Mediated Signalling Pathway, Involving Negative Feedback Inhibition

**DOI:** 10.1371/journal.pone.0026674

**Published:** 2011-11-17

**Authors:** Sakshi Gupta, Kanchan Mishra, Avadhesha Surolia, Kakoli Banerjee

**Affiliations:** 1 Eukaryotic Gene Expression Laboratory, National Institute of Immunology, New Delhi, India; 2 Centre for Molecular Medicine, National Institute of Immunology, New Delhi, India; 3 Molecular Biophysics Unit, Indian Institute of Sciences, Bangalore, India; University of Memphis, United States of America

## Abstract

**Background:**

Suppressors of cytokine signalling (SOCS) protein family are key regulators of cellular responses to cytokines and play an important role in the nervous system. The SOCS6 protein, a less extensively studied SOCS family member, has been shown to induce insulin resistance in the retina and promote survival of the retinal neurons. But no reports are available about the role of SOCS6 in neuritogenesis. In this study, we examined the role of SOCS6 in neurite outgrowth and neuronal cell signalling.

**Methodology/Principal Findings:**

The effect of SOCS6 in neural stem cells differentiation was studied in neural stem cells and PC12 cell line. Highly elevated levels of SOCS6 were found upon neural cell differentiation both at the mRNA and protein level. Furthermore, SOCS6 over-expression lead to increase in neurite outgrowth and degree of branching, whereas SOCS6 knockdown with specific siRNAs, lead to a significant decrease in neurite initiation and extension. Insulin-like growth factor-1 (IGF-1) stimulation which enhanced neurite outgrowth of neural cells resulted in further enhancement of SOCS6 expression. Jak/Stat (Janus Kinase/Signal Transducer And Activator Of Transcription) pathway was found to be involved in the SOCS6 mediated neurite outgrowth. Bioinformatics study revealed presence of putative Stat binding sites in the SOCS6 promoter region. Transcription factors Stat5a and Stat5b were involved in SOCS6 gene upregulation leading to neuronal differentiation. Following differentiation, SOCS6 was found to form a ternary complex with IGFR (Insulin Like Growth Factor-1 Receptor) and JAK2 which acted in a negative feedback loop to inhibit pStat5 activation.

**Conclusion/Significance:**

The current paradigm for the first time states that SOCS6, a SOCS family member, plays an important role in the process of neuronal differentiation. These findings define a novel molecular mechanism for Jak2/Stat5 mediated SOCS6 signalling.

## Introduction

Nervous system function depends on the complex architecture of neuronal networks and this complexity arises from the morphological intricacy that neurons acquire during the course of differentiation [1]. This process of differentiation is regulated by a variety of signalling mechanisms, including growth factors, cytokines, transcription factors and soluble as well as membrane-bound receptors [2] Though several molecules involved in this signalling are now known, how extracellular signals regulate changes in the cytoskeletal arrangement are just beginning to be elucidated. The “Suppressors of Cytokine Signalling” (SOCS) proteins have been shown to be involved in this process of neuronal differentiation [3], [4].

The SOCS family consists of eight members, CIS (Cytokine Inducible SH2-Containing Protein) and the SOCS 1–7 proteins [5], [6]. The SOCS members are localized in the cytoplasm, where they interact with their target proteins [7], [8]. It has been shown that SOCS1, SOCS2 and SOCS3 are all expressed in the nervous system throughout development [9]. SOCS1 regulates the interferon gamma mediated sensory neuron survival [10]. SOCS2 is involved in the neuronal differentiation by inhibiting the growth hormone (GH) signalling and induces neurite-outgrowth by regulation of epidermal growth factor receptor activation [3], [11], [12]. SOCS3 over-expression inhibits astrogliogenesis and promotes maintenance of neural stem cells (NSC) [13], [14]. We have previously shown that SOCS3 is activated by IGF-1 and is also involved in neuronal cell survival and differentiation [15]. *In vitro* studies have implicated insulin-like growth factor-1 (IGF-1) in neuronal differentiation [16]. Mice, carrying a null mutation in the IGF-1 gene display a decrease in cortical thickness while the ventricular zone is enlarged, suggesting that absence of IGF-1 leads to anomaly in the differentiation of stem cells into neurons [17], [18]. Likewise, transgenic mice overexpressing IGF-1 show an enlarged cortex [19].

The SOCS6 protein is a less extensively studied SOCS family member. It has been shown to induce insulin resistance in the retina and promote survival of the retinal neurons [20]. Though it does not inhibit signalling via growth hormone, leukaemia inhibitory factor, or prolactin, it is known to impair the Insulin Receptor signalling and is involved in the proteasome mediated degradation [21]–[28]. Out of all the SOCS family members, SOCS6 has a unique addition of 300 amino acids to its N-terminal region, but the role of this addition remains unclear. Thus the SOCS6 protein might be expected to function differently than the other SOCS members. In this study, we have described a novel role of SOCS6 in neuronal differentiation. We have identified the transcription factors that mediate SOCS6 upregulation in the signalling pathway leading to neurite differentiation.

## Materials and Methods

### Ethics statement

Animal procedures were approved by the National Institute of Immunology's Institutional Animal Ethics Committee.

The Ethics Approval ID number is: IAEC 237/10.

### Reagents and plasmids

IGF-1, IL-6 (Interleukin), TNF-α (Tumor Necrosis Factor-α), mEGF (Murine Epidermal Growth Factor) and bFGF (Basic Fibroblast Growth Factor) were purchased from PeprotechAsia/Cytolab (New Jersey, USA) and PMSF, glutamine and penicillin-streptomycin from Sigma-Aldrich (St. Louis, Missouri, USA). Antibodies against Stat5, SOCS6, pY20 (phosphor-Tyrosine), IGFR, Jak2 and GAPDH were from Santa Cruz Biotechnology (Santa Cruz, California). Antibody against phospho-Stat5 was from Cell Signalling technologies (Danver, MA). Anti-mouse-HRP and anti-rabbit-HRP were from GE Healthcare (Buckinghamshire, UK). Dulbecco's modified Eagle's medium (DMEM), neurobasal media, trypsin-EDTA, B27 supplement, NGF, fetal bovine serum, horse serum, Glutamax, antibiotics and antimycotics agents were from Invitrogen (NY, USA). Protein G Separose beads were procured from GE Healthcare (Buckinghamshire, UK). Protease inhibitors were from Roche Molecular Systems (Alameda, CA, USA). Inhibitors tyrphostin AG490 was from Sigma-Aldrich (St. Louis, Missouri, USA). All other fine chemicals were from Sigma-Aldrich (St. Louis, Missouri, USA). β-galactosidase plasmid was generously provided by Dr S Sengupta, (NII, India), and STAT5A-pRK5, STAT5B- pRK5 and their dominant negatives αSTAT5A- pRK5 and αSTAT5B- pRK5 plasmids were a gift from Dr. James Ihle (Howard Hughes Medical Institute, Memphis, Tennessee).

### Animals

Timed mated Sprague Dawley (Charles River, Sulzfeld, Germany) rats were bred and culled as done previously [15]. Neocortical tissue was dissected from embryonic day 14–16 rat brains and processed as before [15].

### Cell cultures

The protocol for neurosphere culture followed was adapted from a procedure described previously [29]. Neurospheres were prepared from embryonic day 14–16 (E14–16) rat embryo cortex and sub-ventricular zone (SVZ) of day 2 rat pups (P2). Cortices were dissected in neural basal medium supplemented with B27, glutamine, Glutamax, pen-strep and growth factors bFGF and mEGF and the cells were seeded in T-25 flasks and were grown as neurospheres at 37°C in a humidified atmosphere with 5% CO_2_ (Thermo Scientific) so as to obtain neurospheres.

For differentiation, neurospheres were mechanically dissociated and plated on the poly-lysine coated plates without growth factors and cultured for 4–8 days at 37°C in a humidified atmosphere with 5% CO_2_. IGF-1 was used at 20 ng/ml for neurospheres stimulation while preparing lysate.

PC12 (rat pheochromocytoma) cells were cultured in DMEM medium with 5% fetal bovine serum, 10% horse serum, antibiotic, and antimycotic agents (complete growth medium). Cells were differentiated by removal of serum and addition of 50 ng/ml nerve growth factor (NGF; Invitrogen) for 48 hours. The cells were grown in a humidified incubator at 37°C with 5% CO_2_. IGF-1 was used at 50 ng/ml for cells stimulation while preparing lysate.

### Construction of the cDNA for expression of rat SOCS6

Total RNA was isolated from PC12 cells. Total cDNA was prepared using High-Capacity cDNA Archive Kit (Applied Biosystems) as per the manufacturer's instructions. Forward SOCS6 Primer: 5′ CGGAATTCATGAAGAAAATCAGTCTGAA 3′; Reverse SOCS6 Primer: 5′ CGGAATTCTCAGTAGTGCTTCTCCTGCA 3′. The amplified products were cloned into EGFP C1 vector from Clontech.

### Semi-quantitative RT-PCR

Total RNA from neurospheres and differentiated cells were isolated and equal amounts of RNA were reverse trasnscribed into cDNA using High-Capacity cDNA Archive Kit (Applied Biosystems) as per the manufacturer's instructions. The following RT-PCR conditions were employed: SOCS6: 98°C- 1 min, 54.4°C- 1 min 30 sec, and 72°C- 2 min; GAPDH: 94°C- 1 min, 62°C- 1 min, and 72°C- 1 min. Primers used for SOCS6 were the same as those used for cloning rat SOCS6 cDNA. To ensure that the PCR products fall within the linear range, cycle dependency was carried out.

### Transient transfections

PC12 cells were grown to 60% confluence on collagen l coated dishes. DNA was transfected using Lipofectamine 2000 (Invitrogen, NY, USA) as per manufacturer's instructions in OPTI-MEM medium (Invitrogen, NY, USA). The cells were incubated for 6 hours and subsequently the medium was replaced with complete growth medium. The cells were stimulated after 24 hours to 48 hours post transfection.

### Amaxa Transfections

Neuropspheres were grown to in 60 mm dishes. Empty vector EGFP C1, rat SOCS6- EGFP C1 were transfected into the cells using Amaxa nucleofection kit 2000 (Amaxa Corp.) as per the manufacturer's instructions.

### Immunoblot analysis

Cells were plated at a density of 1×10^5^ cells/mL in 60 mm culture dishes and incubated for 24 hours. The cells were serum starved for 12 to 14 hours prior to stimulation with IGF-1 in serum free medium. The media was then aspirated and the cells were washed with ice cold PBS. Subsequently the cells were lysed in ice-cold RIPA buffer containing 100 µg/ml phenylmethylsulphonyl fluoride and 1× protease inhibitor cocktail (Roche, Basel, Switzerland). 50 µg of protein samples were electrophoresed on denaturing SDS-PAGE (Polyacrylamide Gel Electrophoresis) gels and transferred to Immobilon-P membranes (Millipore Corp., Bedford, MA) and probed with antibodies. Immunoreactivity was revealed with horseradish peroxidase-conjugated anti-rabbit or anti-mouse secondary antibodies (GE Healthcare) and enhanced chemiluminescence reagents (GE Healthcare) [15].

### Immunoprecipitation

Cells were plated and subsequently treated as described previously and lysed in RIPA buffer. About 300 µg of lysate was incubated with 1–2 µg of antibodies at 4°C overnight on an end-to-end shaker. Subsequently, antigen-antibody complexes were incubated with 50 µl of protein G-Sepharose beads (GE healthcare) for 2 hours with end-to-end shaking. After washing with lysis buffer 3 times, beads were finally resuspended in 50 µl of sample buffer and blotted as described previously.

### Small interfering RNA–mediated silencing

PC12 cells were cultured for 2–3 days to 60–70% confluency and transfected with siRNA oligonucleotides pool (Dharmacon, Lafayette, CO) using lipofectamine 2000 (Invitrogen Life technologies), according to manufacturer's instructions. Cells were maintained in medium for 4 days before stimulation with agonists. As control, cells received an equal amount of labeled control oligonucleotides (green, non-targeting oligonucleotides from Dharmacon). The effect of antisense oligos was determined by immunoblot analysis and morphological studies.

### Evaluation of neuritogenesis

Number of neurites per cell was counted. Neurite length was measured in randomly chosen cells, in at least n = 3 experiments, essentially as previously described [11]. Cells were visualized and images of all neurons in random fields were captured using a Nikon TE2000 microscope fitted with a CCD camera and appropriate excitation/emission filters. Adobe photoshop and software was used for preparation of images. Neurite lengths were measured by tracing individual neurites using Leica IM50 software (Leica Microsystems Imaging Solutions, Cambridge, UK). Average number of neurite was measured by summing the total number of neurites per differentiated neuron.

### Luciferase assay

1500 bp upstream of ATG of SOCS6 gene was amplified and cloned into pGL3 basic vector from Promega (USA). The chimeric construct was then co-transfected along with pcDNA3.1 Stat1, pcDNA3.1-Stat3, pcDNA3.1- Stat5a, pcDNA3.1- Stat5b, or pcDNA3.1- Stat6 into PC12 cells. As a control for luciferase expression, pGL3 basic vector from Promega (USA) was used. Total amount of DNA/well for all transfections was kept constant at 1.8 µg by co-transfecting the pcDNA3.1 vector. Uniformity of transfection efficiency was achieved by co-transfections with equal quantities of β-Galactosidase vector. After 24 hours, cells were collected and lysed in passive lysis buffer from Promega (USA) followed by β-Galactosidase assay. The luciferase activity in the lysate was measured using a luminometer (Packard LumoCount) Luciferase activity was normalized against β-galactosidase activity to normalize for the variation in transfection efficiency.

### Nuclear and Cytoplasmic Protein Extraction

An extraction kit from Geno-technology (St. Louis, MO) was used to extract proteins from the nucleus and cytoplasm as per the manufacturer's instructions. Briefly, the cells were spun down after treatment, washed with PBS, and resuspended in cytoplasmic extraction buffer. Subsequently, the cells were passed through the narrow mouth tip and centrifuged. The supernatant contained cytoplasmic protein. The pellet was washed several times in the same buffer. Subsequently, nuclear extraction buffer was added and incubated on ice. After centrifugation, the supernatant contained nuclear extract.

### Electrophoretic mobility shift assay (EMSA)

Nuclear and cytoplasmic proteins were isolated as described previously using an extraction kit from Geno-technology (St. Louis, Missouri, USA). To 0.5 µg labeled double stranded oligo-DNA (2×10^5^ cpm),15 µg of nuclear extracts was added and incubated in a 20 µl volume of binding reaction (200 mM HEPES, 4 mM DTT, 50% glycerol, 0.5 µg poly dI:dC, for 30 min at room temperature). In competition experiments, prior to the addition of radioactive probes, 100 fold excess amount of unlabeled competitors were added to the binding reaction and incubated with nuclear extract for 10 min on ice. The binding reaction was then allowed to proceed for 30 minutes at room temperature. All binding mixtures were separated, using 0.5× TBE buffer as the running buffer, at 150 V for 3.5 hours on 4–6% gradient TBE gels. The gels were dried, and analyzed by phosphoimager (Fuji FLA-5000).

### Immunocytochemistry

PC12 cells grown in multiwell Lab-Tek slides were allowed to differentiate for 2 days with NGF (50 ng/ml). Cells were fixed in 3.7% paraformaldehyde, permeabilized in 0.1% Triton, blocked and incubated overnight at 4°C with mouse anti-SOCS6 and rabbit anti-IGFR antibodies. Cells were probed with Alexafluor 594 (Invitrogen) anti-mouse and Alexafluor 488 (Invitrogen) anti-rabbit antibodies. Cells were visualized using Zeiss Axio Imager fluorescence microscope, and images were processed using Adobe Photoshop or Axiovision software.

### Statistical analysis

Data were expressed as means ± S.E. Results were analyzed for statistical significance using *t* test or by ANOVA followed by a Bonferroni Comparison Post Hoc test. All error bars were expressed as SD, with *p<0.05was considered statistically significant difference, **p<0.01 was considered statistically very significant difference and ***p<0.001 was considered statistically extremely significant difference. All the experiments were independently repeated 3 times with similar results.

## Results

### IGF-1 enhances neurite-outgrowth

**Figure 1:**
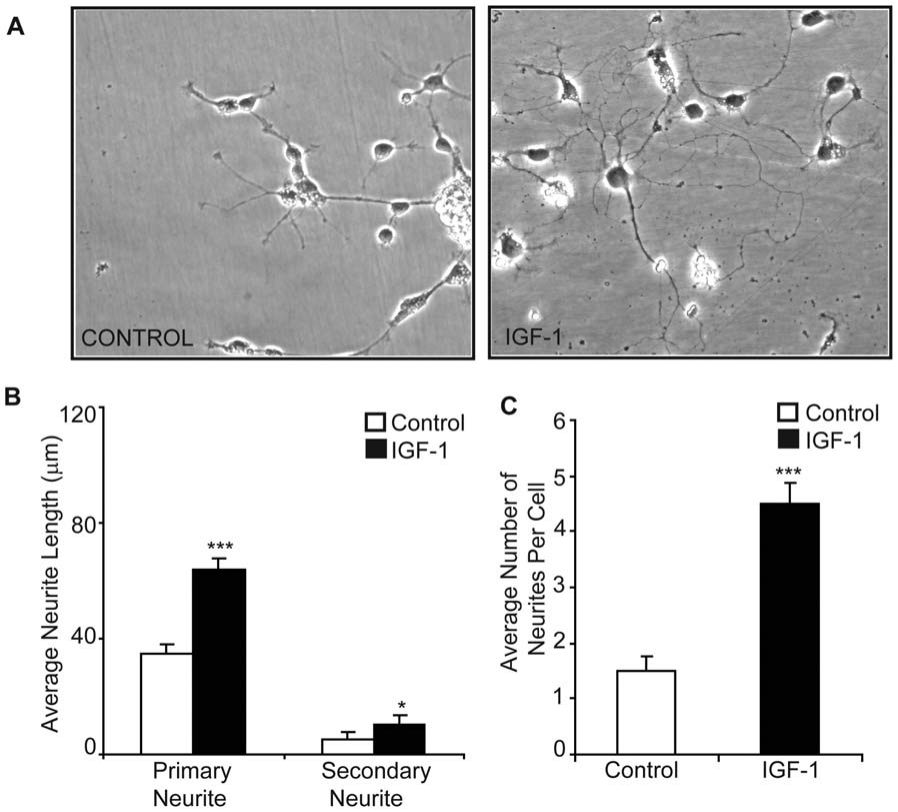
IGF-1 enhances neurite-outgrowth. (**A**) E14 neurospheres were allowed to differentiate in the presence or absence of IGF-1. (**B, C**) Analysis of average of primary and secondary neurite length and average number of neurites per cell in NSCs differentiated in presence and absence of IGF-1. Neurite length was measured in randomly chosen cells (at least 8–10 different fields and approx 5 cells per field) by tracing individual neurites (as described in experimental procedures) and results are expressed as (**B**) average of total primary and secondary neurite lengths or (**C**) average number of neurites per cell. Statistical significance of the difference was determined using ANOVA. The result shows the mean ± S.E. of n = 3 combined experiments (***p<0.001, *p<0.05).

Previously, we have shown that cytokine TNF-α was inhibitory to primary cortical neurons and cell lines, whereas IGF-1 enhanced growth and could rescue cells from TNF-α mediated cell death [15]. In this study the action of exogenous IGF-1 on the differentiation of cortex-derived rat foetal neural stem cells was explored. All experiments were performed in the absence of B-27 as it contained insulin which has similar mechanism of action as IGF-1. Neural stem cells (NSCs) were allowed to differentiate without growth factors, in the presence of IGF-1 for 4 days. Increased neurite-outgrowth was observed in IGF-1 treated cells as compared to control (**Figure 1A**). IGF-1 treated cells had significantly longer length of primary and secondary neurites as well as more number of neurites per cell as compared to the control cells (**Figure 1B and 1C**). Thus IGF-1 promoted neurite-outgrowth as well as branching of foetal NSCs where increased neurite-outgrowth is indicative of differentiation.

### SOCS6 is triggered by differentiation and further enhanced by IGF-1

**Figure 2:**
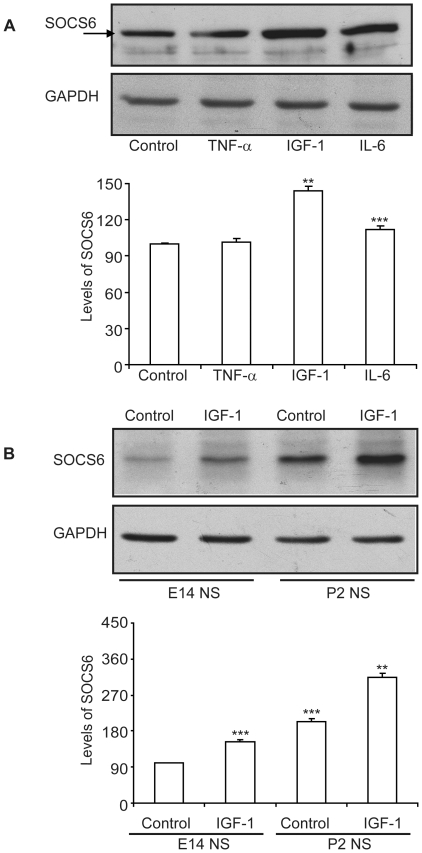
IGF-1 stimulation enhances SOCS6 levels. (**A**) Primary neurospheres were generated from cortex of E14 rat embryos and stimulated with TNF-α (100 pg/ml), IL-6 (20 ng/ml) or IGF-1 (20 ng/ml) for three hours. The cell lysate was immunoblotted with anti-SOCS6 antibody. The same membrane was stripped and reprobed with anti-GAPDH antibody for protein loading control. (**B**) Primary neurospheres were generated from cortex of E14 and sub ventricular zone of pups at postnatal day two (P2) and stimulated with/without IGF-1 for 3 hours. The cell lysate was immunoblotted with anti-SOCS6 antibody. The same membrane was stripped and reprobed with anti-GAPDH antibody for protein loading control. The densitometry data shown was normalized with the untreated control (taken as 100%). The result shows the mean ± S.E. of n = 3 combined experiments (***p<0.001, **p<0.01).

In order to explore the effect of growth factor/cytokine mediated signalling on SOCS family members, neurospheres from cortex of embryonic day 14 (E14) rat embryos were cultured and stimulated with TNF-α, IL-6 and IGF-1. The cells were stimulated for 3 hours and immunoblotting was performed using various anti-SOCS antibodies (**Figure S1**). Among the various SOCS tested, SOCS3 showed a modest increase with IGF-1 and IL-6 whereas SOCS6 expression was found to be upregulated maximally by IGF-1 (**Figure S1 and** **Figure 2A**). There was a 40% increase in SOCS6 levels following IGF-1 stimulation as compared to about 10% with IL-6 (Figure 2A). In order to find out whether SOCS6 expression was restricted only to embryonic cortical stem cells, neurospheres of young pups at postnatal day 2 (P2) were cultured from the sub-ventricular zone; a region where maximum number of NSCs are found in newborn pups. SOCS6 levels were higher at P2 compared to E14 and showed a higher sensitivity to IGF-1 at P2, indicating an increasing requirement of SOCS6 during development (**Figure 2B**).

### Temporal upregulation of SOCS6 upon differentiation

**Figure 3:**
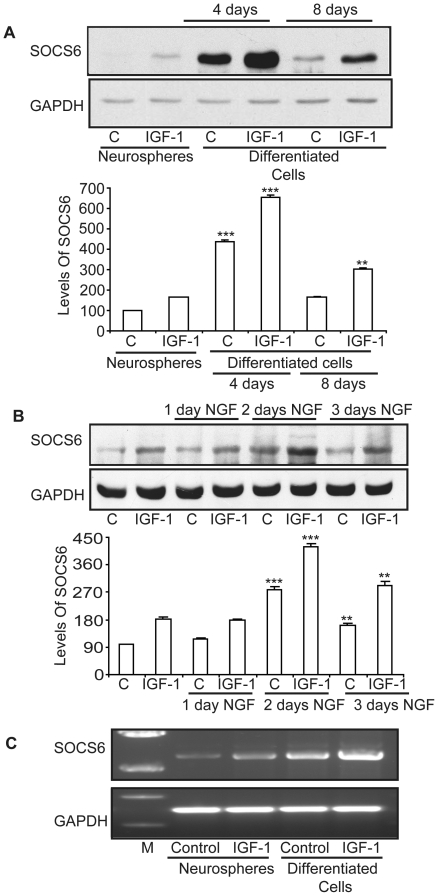
Temporal increase in SOCS6 levels following differentiation. (**A**) Western-blot analysis of SOCS6 expression in E14 neurospheres and neurospheres upon differentiation at day 4 and day 8 with/without 3 hours of IGF-1 (20 ng/ml) stimulation. The same membrane was stripped and reprobed with anti-GAPDH antibody for protein loading control. (**B**). Western-blot analysis of SOCS6 expression in undifferentiated PC12 cells and PC12 cells differentiated with NGF (50 ng/ml) for 1, 2 or 3 days and stimulated with/without IGF-1 for 3 hours. The same membrane was stripped and reprobed with anti-GAPDH antibody for protein loading control. (**C**) E14 neurospheres or neurospheres upon 4 days of differentiation were stimulated with/without IGF-1. Total RNA was isolated and RT-PCR was performed using SOCS6 specific primers and GAPDH primers on the same sample. (M = Marker; C = Control). The result shows the mean ± S.E. of n = 3 combined experiments (***p<0.001, **p<0.01). The densitometry shown below was normalized with the untreated control (taken as 100%).

Upregulation of SOCS6 at P2 stage was indicative of SOCS6 requirement during differentiation. To explore the role of SOCS6 in neural differentiation, neurospheres generated from the embryonic rat-cortex were differentiated following withdrawal of growth factors for 4 and 8 days and then stimulated with IGF-1. The level of SOCS6 in differentiated cells was compared with neurospheres in the presence or absence of IGF-1 by immunoblotting. A 400 fold increase in SOCS6 levels were observed following 4 days of differentiation (**Figure 3A**). Expression pattern was temporal with levels peaking on day 4 of differentiation and reducing thereby. Thus for all subsequent experiments 4 days differentiated NSCs were used. SOCS6 levels were further enhanced by about 45% upon IGF-1 stimulation (**Figure 3A**). To understand the contribution of specific neural cell types towards increase in SOCS6, stem cells were differentiated into various neural subtypes, stained with lineage specific antibody markers and counted (**Figure S2**). Neurons were about 40%, astrocytes about 50% and oligodendrocytes were about 9% after 4 days of differentiation and the difference between neurons and astrocytes was statistically insignificant. There was a marginal increase in the number of astrocytes after 8 days of differentiation. These numbers were very similar to a previous study [30]. Thus the decrease in SOCS6 after 4 days was not due to decrease of any specific neural cell subtype.

To check if SOCS6 expression pattern was similar in non-stem cells, PC12 cells were differentiated for 1, 2 and 3 days with NGF. PC12 has been previously used as an instructive model for studying the underlying mechanisms of neuronal differentiation in response to NGF [31]. SOCS6 levels were elevated in NGF differentiated PC12 cells and further enhanced by about 45% upon IGF-1 stimulation, as compared to undifferentiated cells. SOCS6 expression pattern was again temporally regulated with maximum levels after 2 days of NGF treatment and reducing thereafter (**Figure 3B**). Thus all further experiments were performed using 2 days differentiated PC12 cells.

In order to see if the increase in SOCS6 expression occurred at transcriptional level, neurospheres and neurospheres differentiated for 4 days were stimulated with IGF-1 and the levels of SOCS6 mRNA was determined by reverse transcriptase PCR. An increase in the mRNA levels of SOCS6 was observed in differentiated cells, which was further enhanced by IGF-1 stimulation (**Figure 3C**). These results indicated that increase in SOCS6 expression in differentiated cells occurred at transcriptional level.

### SOCS6 enhances neurite-outgrowth

**Figure 4:**
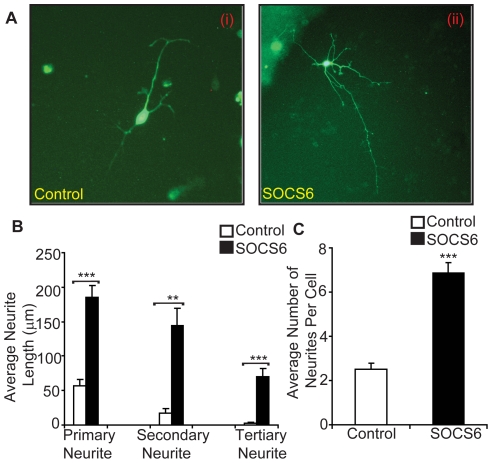
SOCS6 enhances neurite-outgrowth. (**A**) Following withdrawal of growth factors, E14 neurospheres were transiently transfected with control (EGFP vector alone) (i) or SOCS6 (SOCS6-EGFP) (ii) and observed under fluorescent microscope. (**B, C**) Neurite length was measured in randomly chosen cells (at least 6–8 different fields and approx 5 cells per field) by tracing individual neurites (as described in experimental procedures) and results are expressed as (**B**) average of total primary, secondary and tertiary neurite lengths or (**C**) average number of neurites per cell. Statistical significance of the difference was determined using ANOVA. The result shows the mean ± S.E. of n = 3 combined experiments (***p<0.001, **p<0.01).

In order to find out the effect of SOCS6 on neurite-outgrowth and branching, neurospheres were transiently transfected with SOCS6-EGFP or EGFP vector and were allowed to differentiate. SOCS6 transfected cells showed substantially enhanced neurite-outgrowth and branching, as compared to the vector alone cells (**Figure 4A, i and ii**). SOCS6 transfected cells had significantly longer primary, secondary and tertiary neurites as compared to the vector alone cells (**Figure 4B**). Average number of neurites per cell were also much higher in SOCS6 transfected cells as compared to vector alone cells (**Figure 4C**). Results shown are from the counts of EGFP expressing cells, regardless of their level of EGFP expression.

To confirm this novel role of SOCS6 in cell lines, SOCS6 and EGFP expressing stable PC12 cell lines were constructed and differentiated with NGF. SOCS6 transfected cells had augmented neurite-outgrowth as shown by enhanced primary, secondary and tertiary branching as well as higher number of neurites per cell, as compared to the vector alone cells (**Figure S3A and Figure S3B,C**). At any given time point the number of cells with neurites, was similar in both EGFP and SOCS6 stable cells. Thus over-expression of SOCS6 promoted neurite-outgrowth both in NSCs and PC12 cell line.

### Inhibition of neurite-outgrowth by SOCS6 specific siRNA

**Figure 5:**
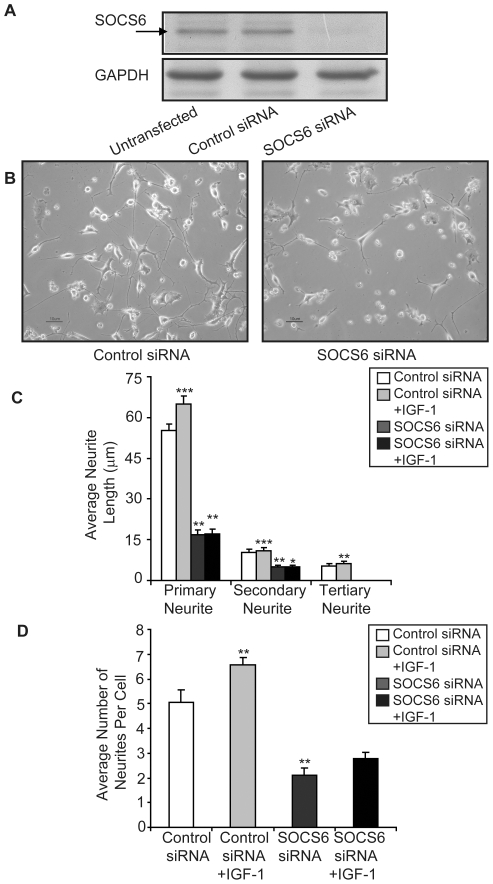
siRNA-mediated silencing of SOCS6 expression inhibits neurite-outgrowth. (**A**) 3 µg of siRNA was transfected into PC12 cells and after 6 days of transfection, the cells were screened for SOCS6 knockdown by Western-blot analysis, using anti-SOCS6 antibody. Control-siRNA (green non-targeting) was used as a negative control. The same membrane was stripped and reprobed with anti-GAPDH antibody for protein loading control. (**B**) Morphology of SOCS6-siRNA transfected PC12 cells as compared to the control transfected cells after 6 days of transfection with NGF treatment under light microscope. (**C and D**) Neurite length was measured in randomly chosen cells (at least 8–10 different fields and approx 5 cells per field) transfected with either control or SOCS6 siRNA with or without IGF-1. Neurite lengths were measured by tracing individual neurites (as described in experimental procedures) and results are expressed as (**C**) average of total primary, secondary and tertiary neurite lengths or (**D**) average number of neurites per cell. Statistical significance of the difference was determined using ANOVA. The result shows the mean ± S.E. of n = 3 combined experiments (***p<0.001, **p<0.01, *p<0.05).

To further confirm the involvement of SOCS6 in promoting neurite-outgrowth, small interfering RNA (siRNA)-mediated silencing of SOCS6 expression was performed. siRNAs were designed against rat SOCS6 gene and transfected into PC12 cells. Standardization of efficient siRNA transfection (about 80%) into PC12 cells was established using control siRNA (green non-targeting) (**Figure S4**). 3 µg of targeting SOCS6-siRNA, knocked out approximately 85% of endogenous SOCS6 protein without noticeable effect on cell viability (**Figure 5A**). As maximum knockdown of SOCS6 protein was observed on day 6, all experiments were performed after 6 days of transfection. Control siRNA had no effect on SOCS6 levels confirming the specificity of SOCS6 siRNA (**Figure 5A**).

In order to investigate the effect of SOCS6 knockdown on neurite-outgrowth, SOCS6-siRNA or control siRNA transfected PC12 cells were differentiated in the presence of NGF. The cells transfected with control siRNA had usual branching pattern and neurite length. In contrast, SOCS6-siRNA transfected cells had fewer branches and smaller neurites (**Figure 5B**). The length of primary neurites in SOCS6-siRNA transfected neurons was reduced with very few secondary and tertiary neurite branching as compared to the cells with control siRNA (**Figure 5C**). Average number of neurites per cell was also much less in SOCS6-siRNA transfected cells as compared to control (**Figure 5D**). IGF-1 stimulation lead to increase in neurite-outgrowth just like in NSC, but its effect was attenuated in SOCS6 depleted cells, indicative of involvement of SOCS6 in IGF-1 mediated differentiation. This data confirms that SOCS6 was involved in neurite-outgrowth which is indicative of differentiation.

### Jak/Stat pathway is involved in SOCS6 upregulation

**Figure 6:**
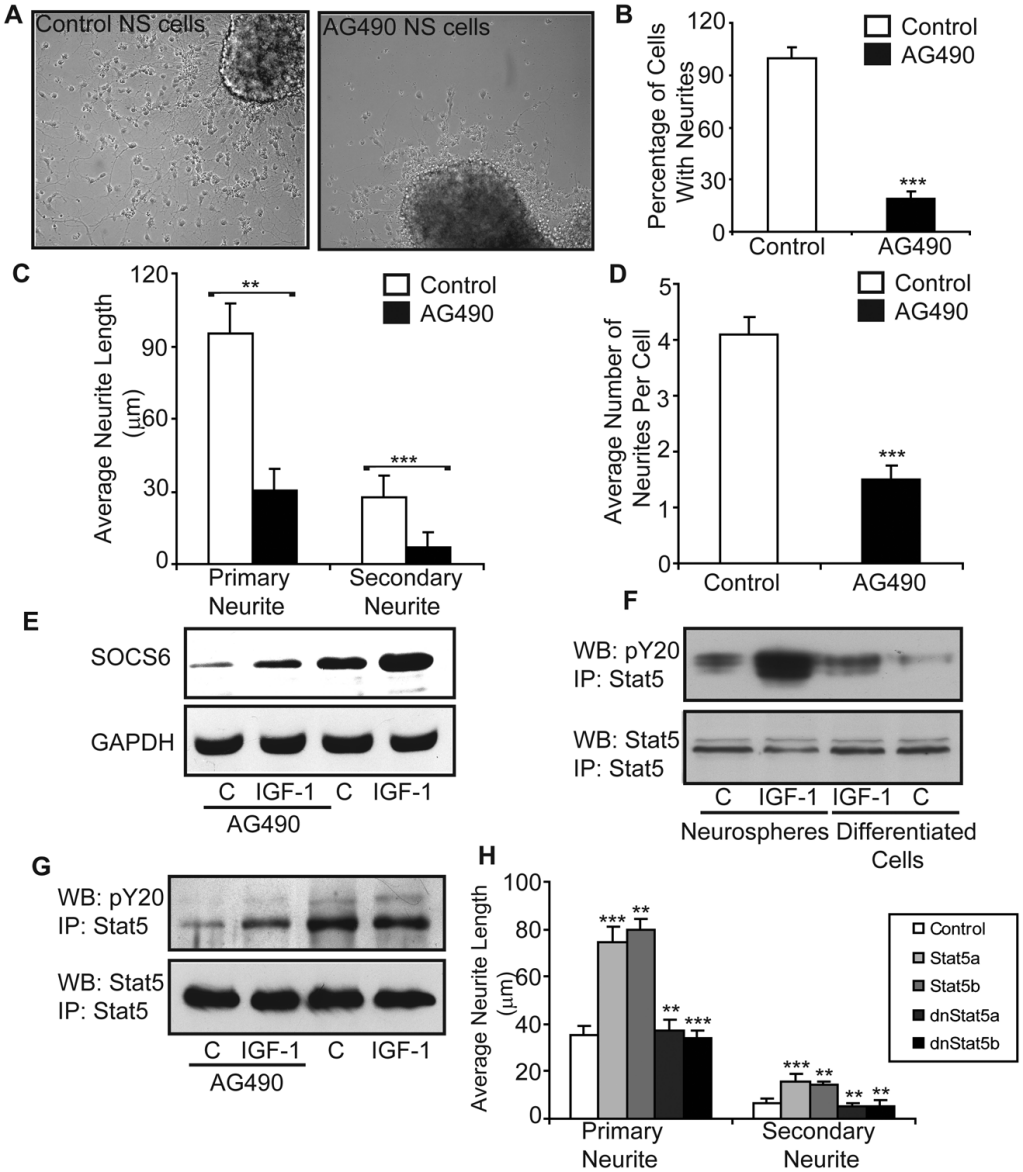
Jak/Stat pathway is involved in neural stem cell differentiation. (**A**) E14 Neurospheres were allowed to differentiate in the absence or presence of 50 µM AG490 (Jak2/Stat3 inhibitor) for 4 days and the cells were observed under light microscope. (**B**) Number of cells with neurites per field was counted. An average of 13 fields was taken. The untreated control was taken as 100%. (**C and D**) Neurite length was measured in randomly chosen cells by tracing individual neurites (as described in experimental procedures) and results are expressed as (**C**) average of total primary and secondary neurite lengths. The untreated control was taken as 100%. (**D**) Average number of neurites per cell. (**E**) Untreated or AG490 treated neurospheres were stimulated with/without IGF-1. The cell lysate was immunoblotted with anti-SOCS6 antibody. The membrane was then stripped and reprobed with anti-GAPDH antibody. (**F**) E14 Neurospheres and neurospheres upon differentiation were stimulated with/without IGF-1 for 10 minutes. Stat5 was immunoprecipitated from 300 µg protein extract and Western-blotted using anti-phosphotyrosine antibody (pY20). The membrane was then stripped and reprobed with anti-Stat5 antibody. (**G**) Untreated or AG490 treated neurospheres were stimulated with/without IGF-1 for 10 minutes. Stat5 was immunoprecipitated from 300 µg protein extract and Western-blotted using anti-phosphotyrosine antibody (pY20). The membrane was then stripped and reprobed with anti-Stat5 antibody. (**H**) PC12 cells, transfected with Stat5a- pcDNA3.1, Stat5b- pcDNA3.1, dominant negative Stat5a- pcDNA3.1 and dominant negative Stat5b- pcDNA3.1 were allowed to differentiate in the presence of NGF and the cells were observed under light microscope. Neurite length was measured in randomly chosen cells by tracing individual neurites (as described in experimental procedures) and results are expressed as average of total primary and secondary neurite lengths. Statistical significance of the difference was determined using ANOVA. The result shows the mean ± S.E. of n = 3 combined experiments (***p<0.001, **p<0.01).

Since SOCS signalling has been shown to involve Jak/Stat pathway, AG490, a pharmacologic inhibitor for the Jak/Stat pathway [32], was used to ascertain its involvement in SOCS6 signalling. When NSCs as well as PC12 cells were treated with 50 µM of AG490, very little neurite-outgrowth and branching was observed in the treated cells as compared to the control cells (**Figure 6A** **and Figure S5A,B**). In both NSCs and PC12 cells, the percentage of cells undergoing differentiation was reduced to almost 20% as compared to the control cells (**Figure 6B** **, Figure S5A,B**). The fewer cells seen in AG490 treated NSCs were due to the fact that the undifferentiated cells did not spread out but remain close to the neurospheres. The length of primary and secondary neurites in the AG490 treated cells was reduced as compared to the untreated control cells (**Figure 6C** **and Figure S5C,D**).). Average number of neurites per cell was also much less in cells cultured in presence of AG490 as compared to the control cells (**Figure 6D** **and Figure S5C,D**). Since 50 µM of AG490 very mildly reduced cell survival (of PC12 cells) as measured by the MTT assay (87.9%±10.3 of controls) (**Figure S5E**), the AG490 inhibitory effect on neurite-outgrowth was unlikely to be a consequence of non-specific cytotoxic effects of AG490. IGF-1 was unable to rescue the inhibitory effects of AG490, indicating that IGF-1 actions were via the Jak/Stat pathway (**Figure S5C and S5C,D**). In order to look for effects of Jak/Stat pathway on SOCS6 expression, NSCs were treated with AG490 with or without IGF-1 stimulation. SOCS6 expression was muted in the presence of the inhibitor (**Figure 6E**), which indicated that SOCS6 was downstream of Jak/Stat. IGF-1 enhanced SOCS6 expression marginally in the presence of the inhibitor, indicating involvement of alternative pathways of SOCS6 stimulation by IGF-1. In order to rule out non-specific action of AG490 on other kinase pathways which could also have an impact on neural cell differentiation, effect of AG490 was checked on erk and PI3 kinase pathways. No non-specific effects were observed in neural stem cells (data not shown). Elevated levels of pStat5 levels were observed in undifferentiated cells as compared to the differentiated cells (**Figure 6F**). IGF-1 was able to substantially enhance pStat5 levels in neuropsheres, but only marginally in differentiated cells, indicating that Jak/Stat5 pathway was more active in neurospheres with its involvement diminishing following differentiation (**Figure 6F**). AG490 which has been previously shown to diminish Stat3 activation also inhibited the activation of Stat5 in NSCs (**Figure 6G**).

To confirm the involvement of Stat5, PC12 cells were transiently transfected with Stat5a, Stat5b, dominant-negative Stat5a (dnStat5a) and dominant-negative Stat5b (dnStat5b) constructs and were allowed to differentiate in the presence of NGF. Stat5a and Stat5b transfected cells showed enhanced neurite-outgrowth and branching, as compared to the vector alone cells (**Figure S6**). In contrast, the cells transfected with dnStat5a and dnStat5b had branching pattern and neurite length comparable to that of control, indicating that Stat5 activated SOCS6 which in turn promoted neurite outgrowth (**Figure 6H**). Together, these data suggest that (a) Jak/Stat was a major pathway for SOCS6 mediated neurite-outgrowth, and (b) differentiation cues inhibit Stat5 activation.

### Stat5 acts as a transcription factor for SOCS6 promoter

**Figure 7:**
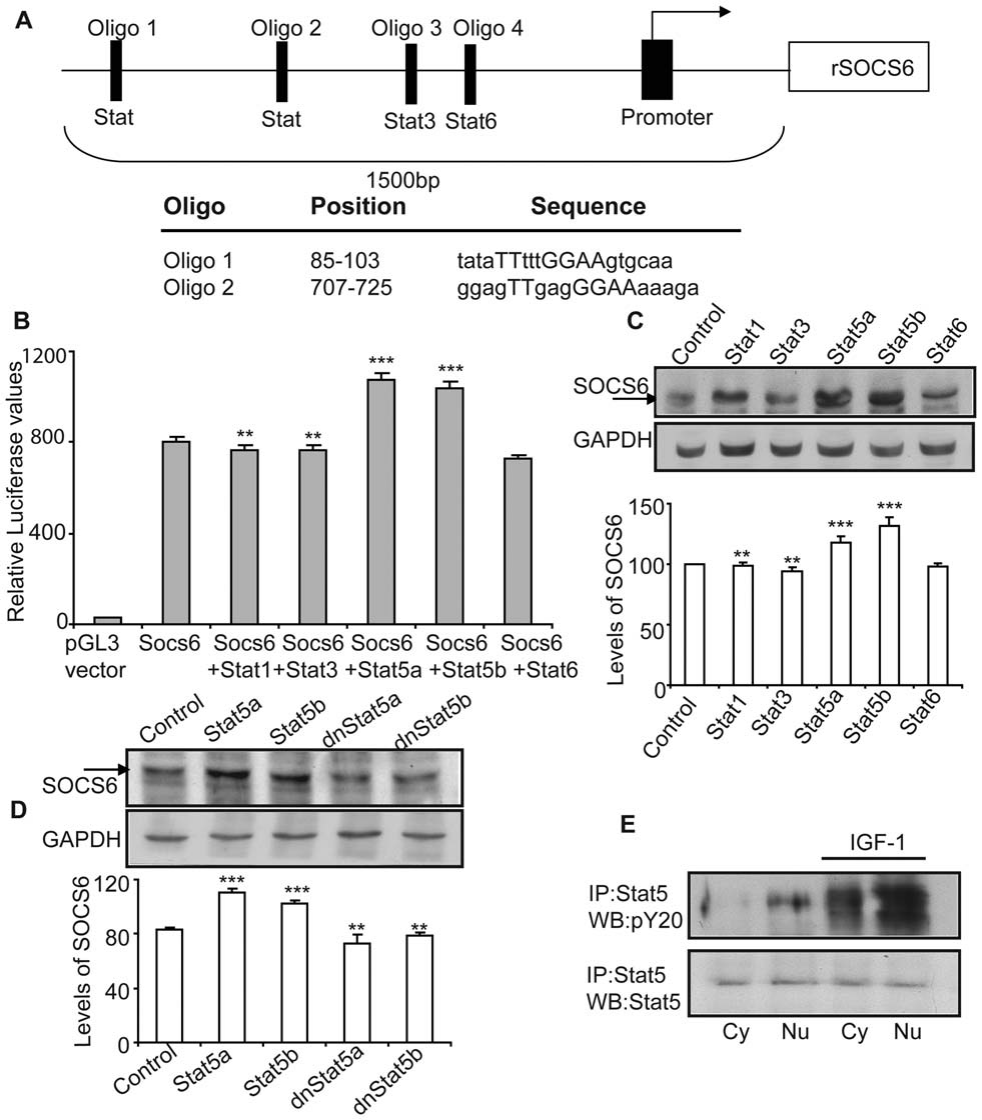
Stat5a and Stat5b upregulate SOCS6 expression. (**A**) Top panel is a diagrammatic representation of the 1500 bp upstream region of rat SOCS6 promoter showing the 2 putative unknown STAT binding sites, one Stat3 and one Stat6 binding sites. Oligo 1 and Oligo 2 are the two oligomers used for the EMSA binding assays. The lower panel displays the sequence of the two oligos based on the Genomatix software predicting the putative STAT binding sites. (**B**) PC12 cells were co-transfected with 1500 bp-pGL3 SOCS6, β-galactosidase plasmid and specific Stat-pcDNA3.1 constructs (Stat1, Stat3, Stat5a, Stat5b, and Stat6). Subsequently a luciferase assay was performed as described in experimental procedures. Each assay was performed in triplicate. (**C**) PC12 cells were transfected with pcDNA3.1-Stat1, pcDNA3.1-Stat3, pcDNA3.1-Stat5a, pcDNA3.1-Stat5b and pcDNA3.1-Stat6. After 2 days, lysates were prepared and the cell lysate was immunoblotted with anti-SOCS6 antibody. The membrane was subsequently stripped and reprobed with anti-GAPDH antibody for protein loading control. (**D**) PC12 cells were transfected with pcDNA3.1-Stat5a, pcDNA3.1-Stat5b, dominant negative pcDNA3.1-Stat5a and dominant negative pcDNA3.1-Stat5b. After 2 days, lysates were prepared and the cell lysate was immunoblotted with anti-SOCS6 antibody and subsequently stripped and reprobed with anti-GAPDH antibody for protein loading control. (**E**) PC12 cells were stimulated with/without IGF-1 and nuclear and cytoplasmic protein was extracted (as described in experimental procedures). Immunoprecipitation was performed with anti-Stat5 antibody and Western-blotted with phosphotyrosine (pY20) antibody. The membrane was stripped and reprobed with anti-Stat5 antibody for loading control. (Cy = Cytoplasmic; Nu = Nuclear). The densitometry shown below was normalized with the untreated control (taken as 100%). The result shows the mean ± S.E. of n = 3 combined experiments (***p<0.001).

To identify the specific Stat(s) involved in SOCS6 upregulation, we selected the 1500 bp TATA less promoter region immediately upstream of SOCS6 start codon and identified various putative Stat binding sites using the Genomatix bioinformatics tool (**Figure 7A**). The 1500 bp upstream region was cloned into pGL3-Basic luciferase expression vector and transfected into PC12 cells either alone or along with various pcDNA3.1- Stat constructs. β-galactosidase plasmid was co-transfected in all cases to normalize the transfection efficiency. Although this 1500 bp genomic region of SOCS6 showed high levels of baseline promoter activity indicating the presence of other factors that could be binding to this region, it was significantly enhanced by Stat5a and Stat5b but not by Stat1, Stat3 or Stat6 (**Figure 7B**). Thus, Stat5 was a likely transactivating factor for SOCS6 gene activation.

To confirm the role of Stat5 in SOCS6 upregulation, PC12 cells were transfected with Stat1, Stat3, Stat5a, Stat5b and Stat6 and SOCS6 expression was checked 24 hours post-transfection. SOCS6 expression levels were highest in Stat5a and Stat5b transfected PC12 cells (**Figure 7C**). To confirm this, dnStat5a and dnStat5b were transfected into PC12 cells. Western-blot analysis showed enhanced levels of SOCS6 in Stat5a and Stat5b transfected PC12 cells whereas dominant negative mutants of Stat5a and Stat5b significantly blocked this activation to almost basal control levels (**Figure 7D**).

Stat5 has been shown to translocate to the nucleus upon stimulation, a response that is inhibited by AG490 [33]. In order to demonstrate the nuclear translocation of activated Stat5 in response to IGF-1 stimulation, PC12 cells were stimulated with or without IGF-1. The nuclear and cytoplasmic fractions were extracted and analyzed for levels of pStat5. pStat5 presence was much more pronounced in both cytoplasmic and nuclear fraction of IGF-1 stimulated PC12 cells with higher levels in the nucleus (**Figure 7E**). This indicated that upon IGF-1 stimulation, there was activation and translocation of pStat5 into the nucleus.

**Figure 8:**
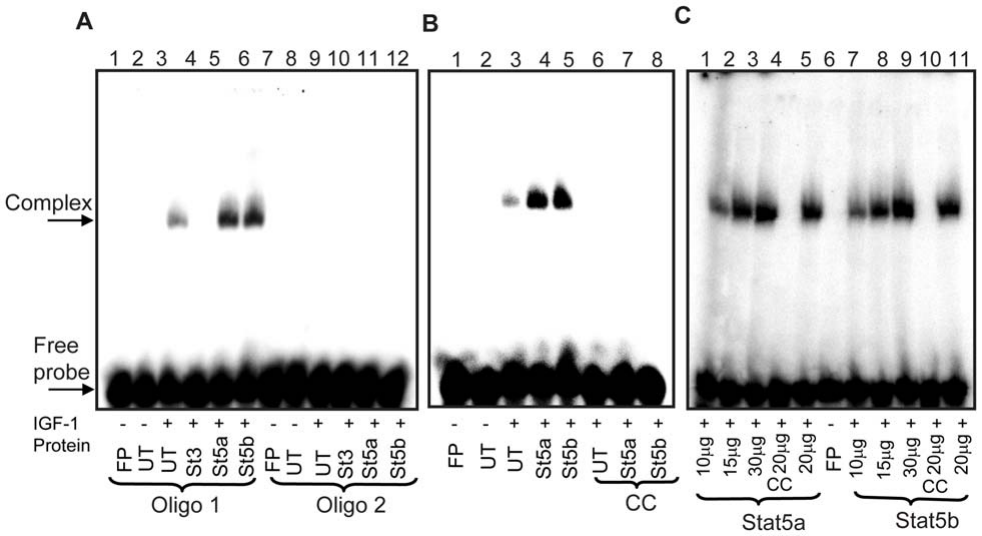
Stat5a and Stat5b are the transcription factors for SOCS6 promoter. (**A**) EMSA with Oligo 1 and Oligo 2 was performed using nuclear extracts from untransfected and PC12 cells transfected with Stat3-EGFP, Stat5a-EGFP and Stat5b-EGFP and stimulated with IGF-1 for 1 hour. Oligo 1 was used in lanes 1–6 and Oligo 2 in lanes 7–12. Lane 1, without cell extracts (free probe); lane 2, negative control (untransfected and without IGF-1 stimulation); lane 3, control (untransfected and with IGF-1 stimulation); lane 4, Stat3-EGFP; lane 5, Stat5a-EGFP; lane 6, Stat5b-EGFP; lane 7, without cell extracts (free probe); lane 8, negative control (untransfected and without IGF-1 stimulation); lane 9, control (untransfected and with IGF-1 stimulation); lane 10, Stat3-EGFP; lane 11, Stat5a-EGFP; lane 12, Stat5b-EGFP. (**B**) EMSA with Oligo1 with cold competition assay. Specificity of binding was checked using excess of cold oligomer as a competitor. Lane 1, without cell extracts; lane 2, negative control (untransfected and without IGF-1 stimulation); lane 3, control (untransfected and with IGF-1 stimulation); lane 4, Stat5a-EGFP; lane 5, Stat5b-EGFP; lanes (6–8), competition assay with excess of cold Oligo 1; lane 6, control (untransfected and with IGF-1 stimulation); lane 7, Stat5a-EGFP; lane 8, Stat5b-EGFP. (**C**) EMSA with Olig 1. Specificity of binding was further checked by using a protein gradient. Lanes 1–5 were Stat5a transfected and lanes 7–11 were Stat5b transfected and IGF-1 stimulated cell extracts. Lane 1, 10 µg protein; lane 2, 15 µg protein; lane 3, 30 µg protein; lane 4, competition assay with cold probe (20 µg protein); lane 5, 20 µg protein; lane 6, without cell extracts; lane 7, 10 µg protein; lane 8, 15 µg protein; lane 9, 30 µg protein; lane 10, competition assay with cold probe (20 µg protein); lane 11, 20 µg protein. The positions of free probe and specific complex are indicated. All the experiments were independently repeated 3 times with similar results. Free probe = FP, untransfected = UT.

In order to confirm the actual Stat5 binding sequence in the SOCS6 regulatory sequence, EMSA was performed using oligonucleotides Oligo 1 and Oligo 2 (sequence shown in Figure 7A) representing both the putative STAT binding sites (**Figure 8A**). Nuclear extracts prepared from IGF-1 stimulated PC12 cells transfected with Stat5a-EGFP and Stat5b-EGFP, were used for binding studies. Extracts from both Stat5a and Stat5b showed mobility shift with Oligo 1 and no binding was observed with Oligo 2, demonstrating the specificity of binding to Oligo 1 STAT binding sequence (**Figure 8A**). Cold competition experiments using 100 fold excess of cold oligomer was able to specifically compete out both Stat5a and Stat5b transfected extracts, indicating the specificity of the DNA-protein interaction (**Figure 8B**). Increasing protein concentrations (10 µg, 15 µg, 20 µg and 30 µg) resulted in corresponding increase in intensity of the protein-DNA complexes further confirming the specificity of the binding (**Figure 8C**). These results provide for the first time insights into the regulation of the SOCS6 gene by Stat5 in the nervous system.

### Interaction of SOCS6 and IGFR occurs in the nucleus

**Figure 9:**
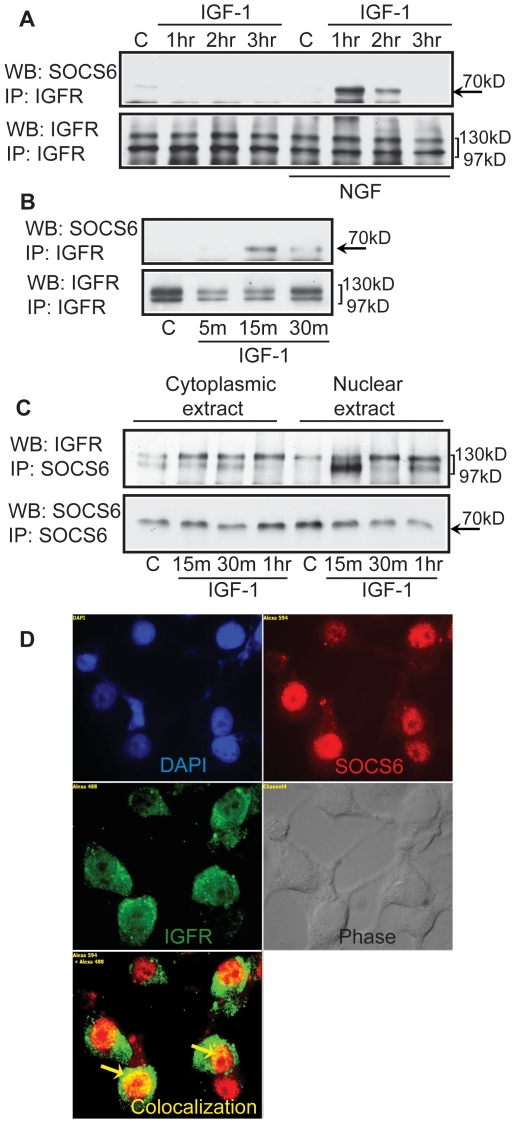
SOCS6 associates with IGFR upon IGF-1 stimulation. (**A**) Undifferentiated or NGF differentiated PC12 cells were stimulated with/without IGF-1 for 1, 2 or 3 hours. Using 300 µg of cell lysate, IGFR was pulled down and Western-blotted with anti-SOCS6 antibody. The membrane was stripped and reprobed with anti-IGFR antibody for loading control. (**B**) SOCS6 stable PC12 cells were stimulated with/without IGF-1 for 5, 15, and 30 minutes. IGFR was immunoprecipitated and Western-blotted with anti-SOCS6 antibody. The membrane was stripped and reprobed with anti-IGFR antibody for loading control. (**C**) PC12 cells were differentiated for 2 days with NGF and then stimulated with/without IGF-1 for 15, 30 m or 1 hr (m = minutes and hr = hours) and nuclear and cytoplasmic protein was extracted (as described in experimental procedures). Using 300 µg of cell lysate, SOCS6 was pulled down and Western-blotted with anti-IGFR antibody. The membrane was stripped and reprobed with anti-SOCS6 antibody for loading control. (**D**) PC12 cells were allowed to differentiate for 2 days with NGF and stimulated with IGF-1 for 30 minutes. The cells were then fixed and permeabilized. After primary antibody treatment (anti-SOCS6, anti-IGFR and anti-SOCS6+anti-IGFR), SOCS6 was stained with Alexafluor 594 (red) and IGFR was stained with Alexafluor 488 (green). The cells were visualized under fluorescent microscope. DAPI staining shows the location of the nucleus.

Previously we have shown that SOCS3 associated with IGF receptor (IGFR) upon IGF-1 stimulation [15]. Also insulin receptor has been shown to associate with SOCS6 upon insulin stimulation [26], but so far no association has been shown for SOCS6 and IGFR. In order to find out if SOCS6 interacted with IGFR, undifferentiated and 2 days differentiated PC12 cells were stimulated with IGF-1 for 1, 2 and 3 hours. IGFR was immunoprecipitated, blotted and probed with anti-SOCS6 antibody. A temporal increase in the association of SOCS6 and IGFR was seen, peaking at 1 hour post IGF-1 stimulation and declining thereafter (**Figure 9A**). This peak coincided with the maximal SOCS6 expression observed on 2 days differentiated cells.

To understand the kinetics of this association, immunoprecipitation studies were performed with PC12 cell lines stably transfected with SOCS6, following varying lengths of IGF-1 stimulation. Association of SOCS6 with IGFR occurred as early as 15 minutes post IGF-1 stimulation (**Figure 9B**). In order to physically locate the association, nuclear and cytoplasmic extracts of IGF-1 stimulated SOCS6 stable-PC12 cells were immunoprecipitated with anti-SOCS6 and blotted with anti-IGFR antibodies. An association between IGFR and SOCS6 was observed within 15 minutes of stimulation as seen before, mostly in the nucleus (**Figure 9C**). To confirm this observation, co-localization studies of IGFR and SOCS6 was performed in PC12 cells differentiated for 2 days. The cells were stimulated with IGF-1 for 30 minutes and immunofluorescence was done using anti-SOCS6 and anti-IGFR antibodies. Secondary antibodies were fluorescently labelled with green (IGFR) and red (SOCS6) dyes and observed under fluorescent microscope. IGFR appeared to be mostly in the cytoplasm but also uniformly distributed within the nucleus but SOCS6 was primarily in the nucleus (**Figure 9D**). Interestingly the association of IGFR and SOCS6 mostly occurred within the nucleus. Control immunofluorescence experiments in the absence of IGF-1 did not show the co-localization of IGFR and SOCS6 in the nucleus (**Figure S7**), indicating that differentiation cues (IGF-1 in this case) are required for IGFR and SOCS6 to interact.

### Feedback inhibition of Jak2/Stat5 pathway occurs via formation of a Jak2-IGFR-SOCS6 complex

**Figure 10:**
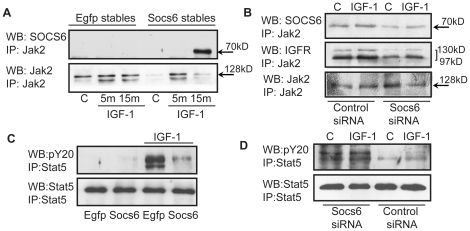
Feedback inhibition of Jak2/Stat5 pathway occurs via formation of a Jak2-IGFR-SOCS6 complex. (**A**) PC12 cells stably transfected with SOCS6-EGFP or EGFP vector alone were stimulated with IGF-1 for 0 m, 5 m and 15 m (m = minute). Jak2 was immunoprecipitated and Western-blotted with anti-SOCS6 (top panel) or anti-Jak2 (bottom panel) antibodies. (**B**) PC12 cells transiently transfected with control or SOCS6-siRNA was stimulated with IGF-1 for 15 minutes. Jak2 was immunoprecipitated and Western-blot was performed using anti-IGFR antibody. The membrane was subsequently stripped and reprobed with anti-SOCS6 and anti-Jak2 antibodies. (**C**) PC12 cells stably transfected with SOCS6-EGFP or EGFP vector alone were stimulated with IGF-1 for 10 minutes. Stat5 was immunoprecipitated and Western-blot was performed using pY20 antibody. The membrane was subsequently stripped and reprobed with anti-Stat5 antibody. (**D**) PC12 cells transiently transfected with control or SOCS6-siRNA was stimulated with IGF-1 for 10 minutes. Stat5 was immunoprecipitated and Western-blot was performed using pY20 antibody. The membrane was subsequently stripped and reprobed with anti-Stat5 antibody. All the experiments were independently repeated 3 times with similar results.

Previously SOCS has been shown to inhibit Jak kinases [34]. In our studies SOCS6 was not found to be associated with Jak1 (data not shown). But co-immunopreciptation of Jak2 and SOCS6 in SOCS6-stable-PC12 cells within 15 minutes of IGF-1 stimulation, demonstrated an association of SOCS6 with Jak2 in response to IGF-1 (**Figure 10A**). The timing of association was similar to SOCS6 with IGFR, indicating the formation of a Jak2-IGFR-SOCS6 complex. In order to reconfirm this association, co-immunopreciptation of Jak2, SOCS6 and IGFR was done in SOCS6-siRNA transfected PC12 cells following 15 minutes of IGF-1 stimulation using anti-Jak2 antibody. As expected SOCS6 and IGFR co-immunoprecipitated with anti-Jak2 antibody, with expression levels of both IGFR and SOCS6 less in SOCS6 siRNA transfected samples as compared to control (**Figure 10B**). In order to find out if the Jak2-IGFR-SOCS6 complex could inhibit subsequent activation of Stat5, EGFP and SOCS6-stable-PC12 cells were stimulated with IGF-1 for 10 minutes. pStat5 levels were lower in SOCS6-stable-cells, thereby indicating that SOCS6 over-expression led to feed-back inhibition of pStat5 (**Figure 10C**). Next the expression of pStat5 was checked in SOCS6 siRNA transfected samples and as expected, inhibiting the expression of SOCS6 resulted in the activation of the pStat5 expression levels (**Figure 10D**). These results confirmed the hypothesis that SOCS6 regulates the Jak2/Stat5 pathway by feed-back mechanism.

## Discussion

Signalling mechanism controlling neuronal differentiation processes has not been well studied and is likely to be a complex process, requiring interplay of many signalling events. We have previously shown that IGF-1 upregulated the expression of SOCS3, which further promoted neurite-outgrowth in primary cortical neurons [15]. In this study, our focus was to identify if any other members of the SOCS family responsive to IGF-1, were involved in neural stem cell differentiation. Our initial studies demonstrated that IGF-1 enhanced the neurite-outgrowth and differentiation of foetal NSCs as shown previously in other cell types [35]–[37]. In neurospheres, SOCS6 levels increased by 30% following IGF-1 stimulation. Interestingly SOCS6 jumped 400 fold following differentiation cues, both at the transcriptional as well as translational levels. IGF-1 further enhanced SOCS6 levels by about 30–40%. Though SOCS6 has been previously shown to be involved in regulation of glucose metabolism, previous studies on SOCS6 knockouts [24] and SOCS6 transgenics [25] have not explored its role in neuronal differentiation or brain development. This is the first time the involvement of SOCS6 in neuronal differentiation has been demonstrated.

NGF treated PC12 cells which have previously been shown to cease proliferation, extend neurites and acquire a number of properties characteristic of sympathetic neurons [38], showed a temporal increase of SOCS6 levels following differentiation cues. Previously only SOCS2 has been shown to regulate NSC differentiation following growth hormone stimulation [39]. NSCs transiently transfected with SOCS6-EGFP plasmids as well as PC12 cell line stably expressing SOCS6 under differentiating conditions, showed increased numbers of neurites, enhanced neurite-outgrowth and longer neurites, all of which are known indicators of differentiation [40]. Unlike an earlier study, no apoptotic effects were seen in SOCS6 overexpressing NSCs and PC12 cells [41]. Further, SOCS6 silencing inhibited neurite initiation and branching, confirming the role of SOCS6 in differentiation.

Since we had previously seen the involvement of Jak/Stat pathway in SOCS3 mediated signalling [15], attempts were made to find out if SOCS6 signalling was also mediated via the Jak/Stat pathway. AG490 a potent inhibitor of Jak/Stat [32], dampened the SOCS6 expression in both NSCs and PC12 cells. Jak/Stat pathway was found to be involved in enhancing the neurite length, number of neurites and branching per cell. This is consistent with the earlier reports where Jak/Stat pathway has been shown to be involved in regulating differentiation of multipotent NSCs into astrocytes and SOCS mediated signalling [42]–[45]. Further, IGF-1 which enhanced SOCS6 expression was unable to rescue the inhibitory effects of AG490, indicating the involvement of Jak/Stat pathway in IGF-1 mediated signalling. Silencing SOCS6 rendered IGF-1 incapable of exerting its effects, which confirmed that IGF-1 action is mediated via SOCS6. Once SOCS6 was activated following differentiation cues, IGF-1 was not required for SOCS6 mediated neurite-outgrowth.

Since the role of Stats as transcription activators of SOCS gene has been reported previously [46]–[48] search for Stat binding sites in the SOCS6 gene promoter revealed various putative Stat binding sites. Increased SOCS6 expression following overexpression of Stat5a and Stat5b, which was inhibited in the presence of dominant–negative Stat5a and Stat5b, was indicative of the role of Stat5 as a transcription factor for SOCS6 gene expression. Stat5 binding site on the SOCS6 promoter region was further confirmed by mobility shift experiments. Though GH has been shown to signal via the Jak2/Stat5b to enhance expression of CIS and SOCS1-3, to our knowledge, this is the first report that demonstrates that Stat5 acts as a transcription factor for SOCS6 expression.

Activation of Stat5 following IGF-1 stimulation in neuronal cells and enhanced neurite outgrowth following Stat5 over-expression provided the *in vivo* evidence of the role of Stat5 in neuritogenesis and neuronal differentiation. Interestingly once the differentiation had occurred there was inhibition of pStat5, an indication of negative feedback. Previous studies have established that Stat5 was important for forebrain and spinal cord development and was inhibited on SOCS2 mediated differentiation [49], [4]. Stat5 has also been shown to be involved in Th2 and hematopoietic cell differentiation [50]–[52]. Stat5 mutants have confirmed its and its role in developing CNS, neuroprotection and neurosignalling [49], [53], [54]. Though it has been shown that IGF-1 activated Stat3 [55], [15], there have been no reports of Stat5 activation by IGF-1. Thus Stat5 was a key mediator for SOCS6 expression and SOCS6 mediated neurite-outgrowth.

IGF-1 stimulation of SOCS6 expression, implicated the involvement of IGF receptor (IGFR) in IGF-1 mediated SOCS6 signalling. Association of SOCS molecules with the cytoplasmic domain of the IGFR has been demonstrated previously [56]. SOCS1 and SOCS2 were found to interact with the IGFR following stimulation with IGF-1 [57], whereas SOCS3 was found constitutively bound to the IGFR [58]. In this study SOCS6 was found to rapidly associate with IGFR upon IGF-1 stimulation, in SOCS6 overexpressing cells. This association was predominantly localized in the nucleus. This was consistent with previous studies showing that receptor tyrosine kinases translocated into nucleus to promote transcription [59]. Moreover IGFR specifically has been shown to translocate into nucleus of neurons, oligodendrocytes and glia [60]. To our knowledge this is the first report that demonstrates the association of SOCS6 with IGFR in the brain.

**Figure 11:**
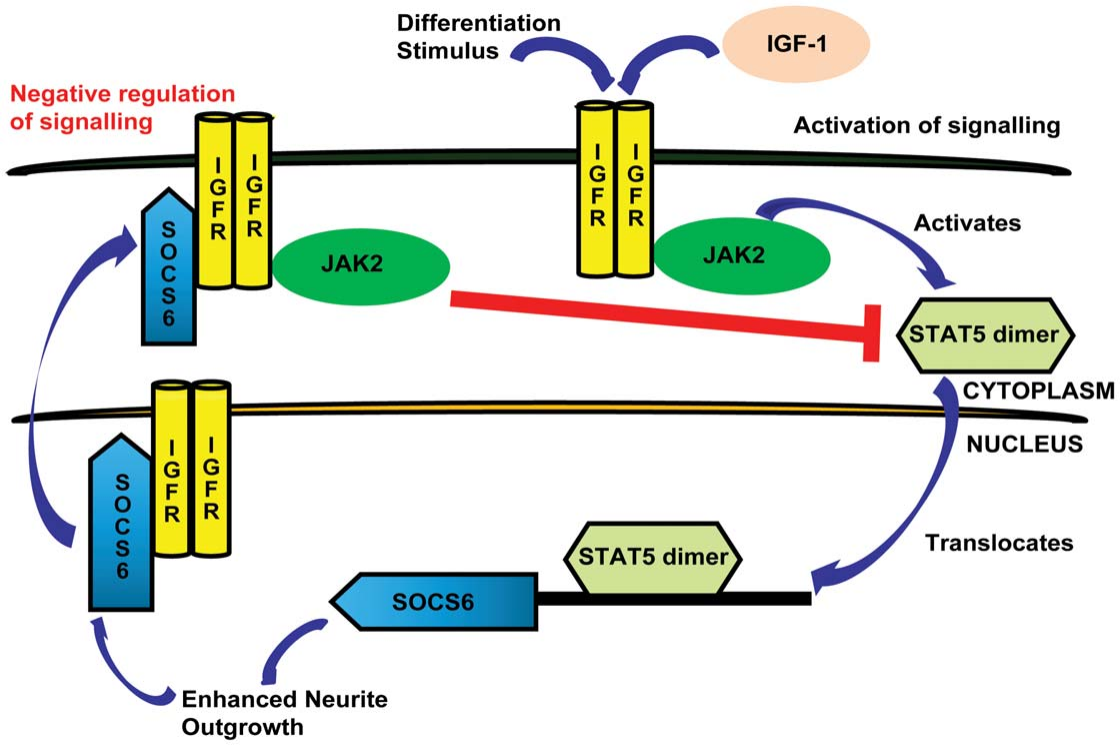
Schematic representation of SOCS6 mediated neural differentiation. Upon IGF-1 stimulation of neural cells, Jak2 is activated which then activates Stat5. Phosphorylated Stat5 dimerizes, translocates to the nucleus and acts as a transcription factor for SOCS6 which in turn promotes differentiation. SOCS6 then binds to IGFR and forms a trio complex with Jak2 leading to the inhibition of further Jak2-Stat5 signalling.

SOCS is known to function in a classical feedback loop by attenuating the signal of its specific activating Stat [61]. Both Jak2 and IGFR was found to be associated with SOCS6 following IGF-1 stimulation in SOCS6 overexpressing PC12 cells. This complex could lead to inhibition of IGF-1 stimulated pStat5 activation, which fits the classical feedback model. Over-expressed SOCS family members like CIS and SOCS1-3, have been shown to interfere with the Jak2/Stat5b pathway [8]. IGF-1 stimulated activation of Stat5 leading to SOCS6 over-expression, followed by negative feedback via Jak2-IGFR-SOCS6 complex, seems to follow a pattern similar to GH signalling [4]. The feedback inhibition could play an important role in restoring cellular responsiveness to subsequent IGF-1 stimulation. Thus, SOCS6 is not only capable of self-tuning its own response but also limit the actions of other SOCS protein family members [62]. This cross-inhibitory function of SOCS6 could be helpful in negating the counter-differentiating properties of other SOCS molecules, just like SOCS2 which is known to bind to SOCS1 and SOCS3 and push it towards proteasome-degradation [62]. The entire signalling mechanism of SOCS6 action following IGF-1/differentiation is depicted diagrammatically in **Figure 11**.

In conclusion, differentiation cues lead to upregulation of SOCS6 which in turn lead to increased neurite-outgrowth. The differentiation process was further enhanced by IGF-1 which was capable of regulating and being regulated by SOCS6. IGF-1 stimulated Stat5a and Stat5b activation, leading to SOCS6 upregulation. Interaction between SOCS6 and IGFR occurred mostly in the nucleus. In a classical negative feedback mechanism SOCS6 was bound to IGFR as well as Jak2, leading to inhibition of Stat5 mediated signalling. Our studies represent the first step towards understanding the role of SOCS6 in neural cell differentiation. While further studies are required to understand the molecular mechanisms of SOCS6 enhanced neural differentiation, these studies show that due to its integral role in the regulation of neural differentiation, SOCS6 could be an important therapeutic target for both the repair of neuronal injuries and treatment of neurological diseases.

## Supporting Information

Figure S1 **Expression pattern of various SOCS proteins following stimulation with TNF-α, IGF-1 and IL-6.** Neurospheres were treated with/without TNF-α, IGF-1 or IL-6 for 3-hours. The cell lysate was immunoblotted with different anti-SOCS antibodies. Subsequently the membrane was stripped and reprobed with anti-GAPDH antibody.
(JPG)

Figure S2 **Neural cell lineages following neural stem cell differentiation.** Neural stem cells were differentiated after the withdrawal of growth factors up to 4 days, immnunofluorescence was done using β-III Tubulin, GFAP and CNPase antibodies for neurons, astrocytes and oligodendrocytes respectively. DAPI was used for nuclear staining. Graph was prepared based on the percentage of different cells types counted. The result shows the mean ±SE of n = 3 combined experiments (***p<0.001, **p<0.01).
(JPG)

Figure S3 **Effect of SOCS6 on neuritic outgrowth of PC12 cells.** (**A**) PC12 cells stable transfected with EGFP or SOCS6 EGFP were allowed to differentiate in the presence of NGF and observed under fluorescent microscope. (**B, C**) PC12 cells stably transfected with EGFP or with SOCS6-EGFP were differentiated with NGF in the presence and absence of IGF-1. Average length of primary, secondary and tertiary branches in EGFP and SOCS6 stably transfected PC12 cells and average number of neurites per cell. Neurite length was measured in randomly chosen cells. Statistical significance of the difference was determined using ANOVA. The result shows the mean ±SE of n = 3 combined experiments (**p<0.01, *p<0.05).Neurite lengths were measured by tracing individual neurites (as described in experimental procedures) and results are expressed as (**B**) average of total primary, secondary and tertiary neurite lengths or (**C**) average number of neurites per cell. Scale bar 5 µM.
(PPT)

Figure S4 **Efficiency of siRNA transfections.** Optimization of efficient siRNA transfection in the PC12 cells using control siRNA (green non-targeting siRNA). This figure shows the merge image of siRNA transfected cells and all the cells present in the well.
(JPG)

Figure S5 **Effect of AG490 on neuritic outgrowth of PC12 cells.** PC12 cells were allowed to differentiate in the presence of 50 µMAG490 (Jak2/Stat3 inhibitor) for 4 days. (**A**) The cells were seen under light microscope. (**B**) Number of cells with neurites per field was counted. An average of 13 fields was taken. The untreated control was taken as 100%. (**C and D**) Neurite length was measured in randomly chosen cells. Statistical significance of the difference was determined using ANOVA. The result shows the mean ±SE of n = 3 combined experiments (**p<0.01, *p<0.05). Neurite lengths were measured by tracing individual neurites (as described in experimental procedures) and results are expressed as (**C**) average of total primary and secondary neurite lengths or (**D**) average number of neurites per cell. Scale bar, 5 µM. (**E**) PC12 cells were allowed to grow in the presence of increasing the concentration of AG490 (0 µM-100 µM) and MTT assay was performed.
(PPT)

Figure S6 **Effect of Stat5 and dominant-negative Stat5 on neuritic outgrowth of PC12 cells.** PC12 cells transfected with pcDNA3.1, pcDNA3.1-Stat5a, pcDNA3.1-Stat5b, pcDNA3.1-dnStat5a or pcDNA3.1-dnStat5bwere allowed to differentiate in the presence of NGF and observed under microscope.
(JPG)

Figure S7 **Background immuno-staining of PC12 cells in the absence of IGF-1 stimulation.** PC12 cells were allowed to differentiate for 2 days with NGF. The cells were then fixed and permeabilized. After primary antibody treatment (anti-SOCS6, anti-IGFR and anti-SOCS6+anti-IGFR), SOCS6 was stained with Alexafluor594 (red) and IGFR was stained withAlexafluor488 (green). The cells were visualized under fluorescent microscope. DAPI staining shows the location of the nucleus.
(JPG)
