# Supplementary figures and images for: Suppressor of Cytokine Signalling-6 Promotes Neurite Outgrowth via JAK2/STAT5-Mediated Signalling Pathway, Involving Negative Feedback Inhibition
Source: PLoS One. 2011 Nov 17;6(11):e26674. doi: 10.1371/journal.pone.0026674 (PMC3219632; doi:10.1371/journal.pone.0026674)

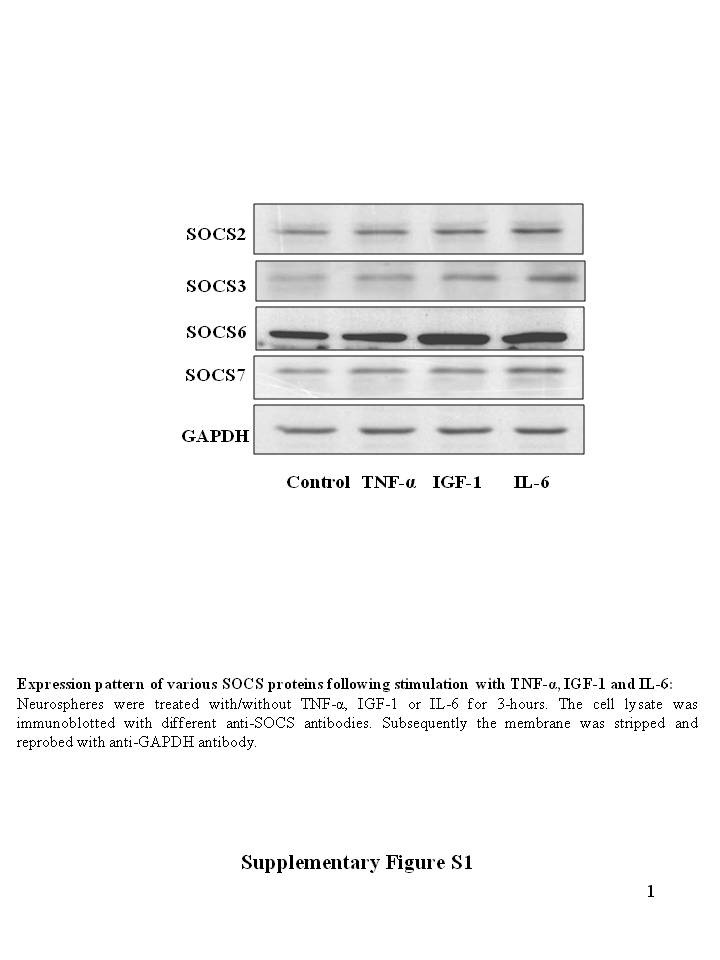

Supplement: Figure S1 — Expression pattern of various SOCS proteins following stimulation with TNF-α, IGF-1 and IL-6. Neurospheres were treated with/without TNF-α, IGF-1 or IL-6 for 3-hours. The cell lysate was immunoblotted with different anti-SOCS antibodies. Subsequently the membrane was stripped and reprobed with anti-GAPDH antibody. (JPG) [file pone.0026674.s001.jpg]

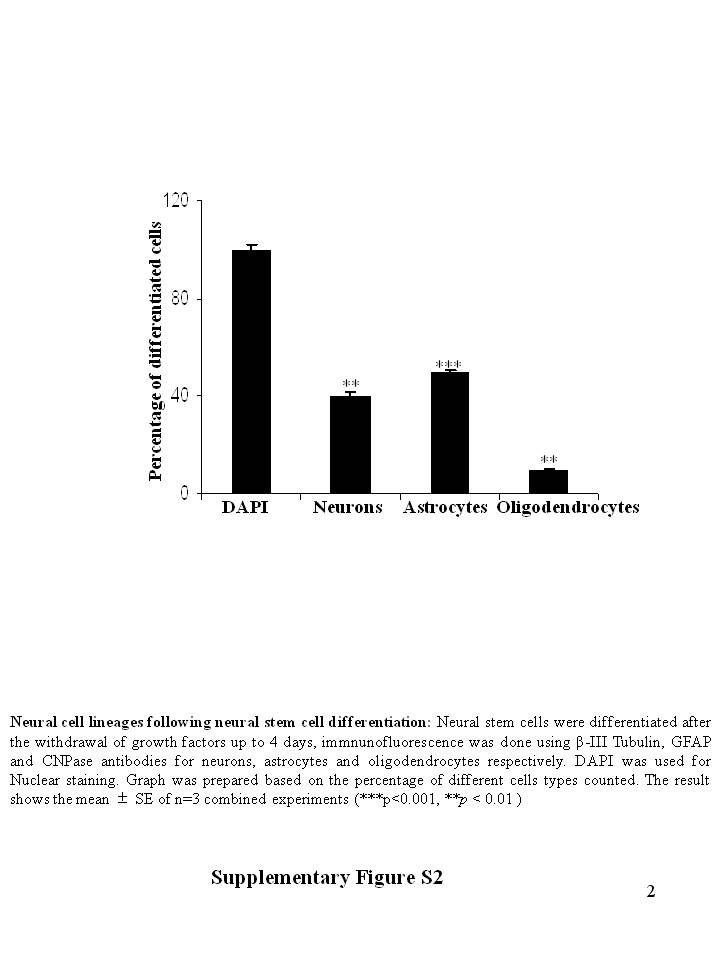

Supplement: Figure S2 — Neural cell lineages following neural stem cell differentiation. Neural stem cells were differentiated after the withdrawal of growth factors up to 4 days, immnunofluorescence was done using β-III Tubulin, GFAP and CNPase antibodies for neurons, astrocytes and oligodendrocytes respectively. DAPI was used for nuclear staining. Graph was prepared based on the percentage of different cells types counted. The result shows the mean ±SE of n = 3 combined experiments (***p<0.001, **p<0.01). (JPG) [file pone.0026674.s002.jpg]

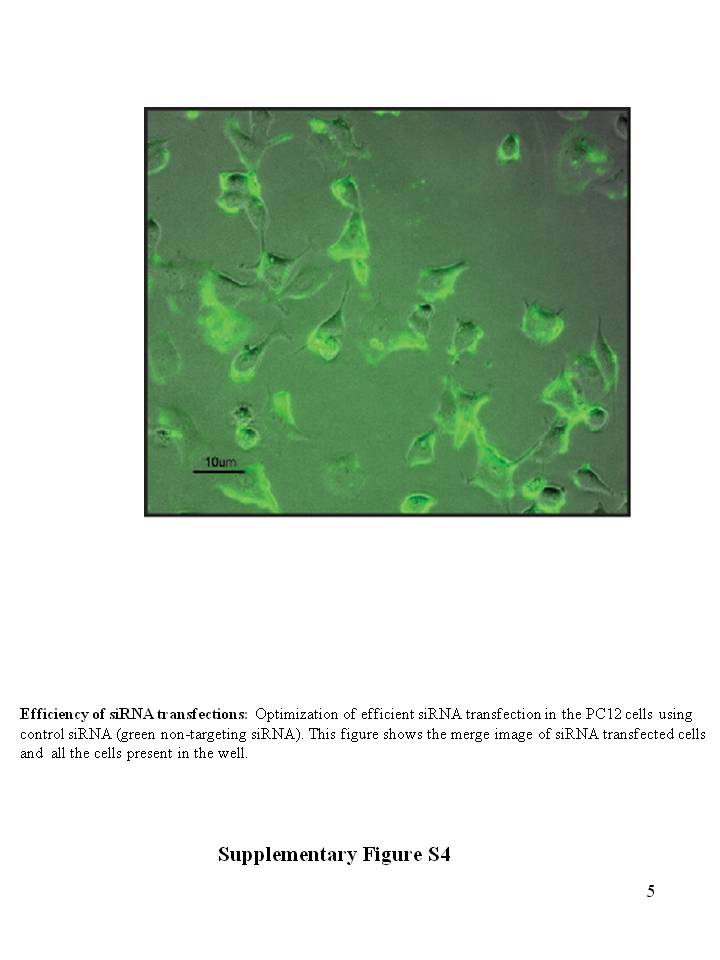

Supplement: Figure S4 — Efficiency of siRNA transfections. Optimization of efficient siRNA transfection in the PC12 cells using control siRNA (green non-targeting siRNA). This figure shows the merge image of siRNA transfected cells and all the cells present in the well. (JPG) [file pone.0026674.s004.jpg]

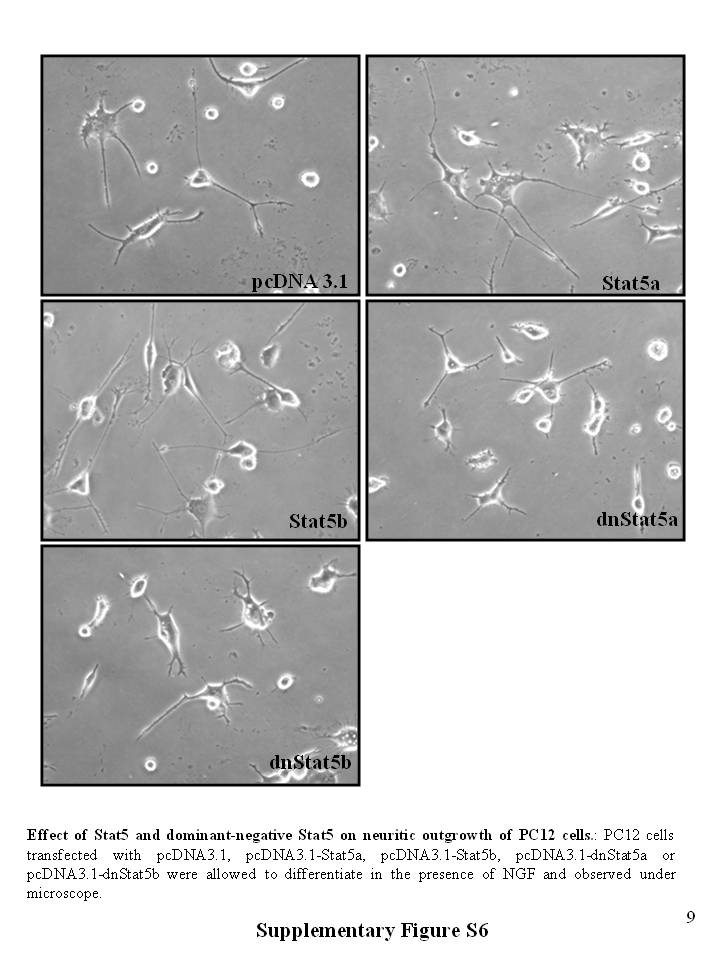

Supplement: Figure S6 — Effect of Stat5 and dominant-negative Stat5 on neuritic outgrowth of PC12 cells. PC12 cells transfected with pcDNA3.1, pcDNA3.1-Stat5a, pcDNA3.1-Stat5b, pcDNA3.1-dnStat5a or pcDNA3.1-dnStat5bwere allowed to differentiate in the presence of NGF and observed under microscope. (JPG) [file pone.0026674.s006.jpg]

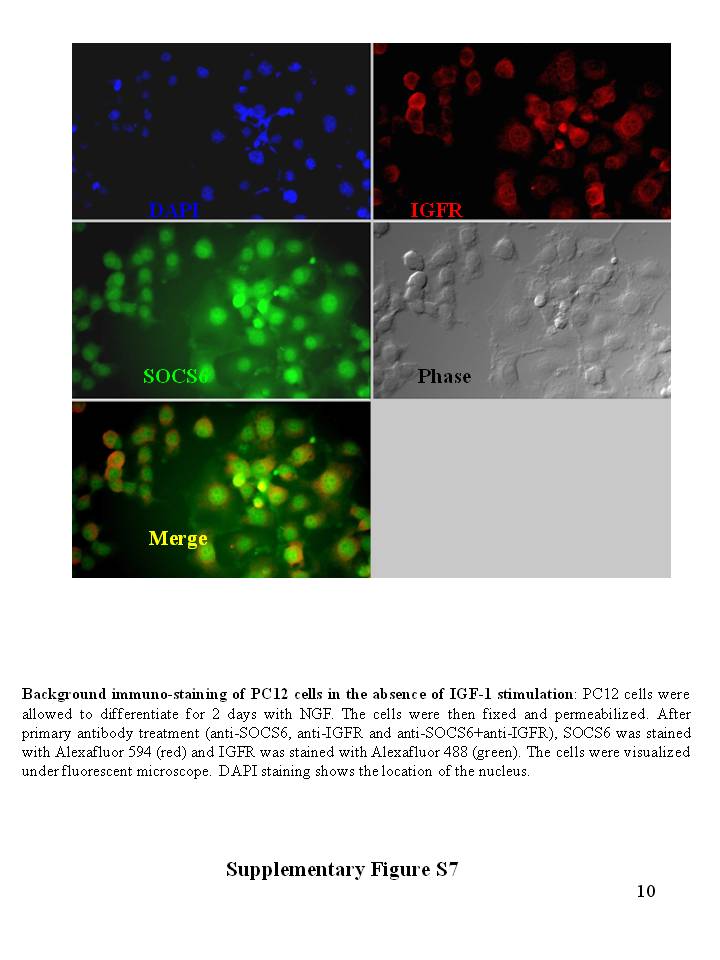

Supplement: Figure S7 — Background immuno-staining of PC12 cells in the absence of IGF-1 stimulation. PC12 cells were allowed to differentiate for 2 days with NGF. The cells were then fixed and permeabilized. After primary antibody treatment (anti-SOCS6, anti-IGFR and anti-SOCS6+anti-IGFR), SOCS6 was stained with Alexafluor594 (red) and IGFR was stained withAlexafluor488 (green). The cells were visualized under fluorescent microscope. DAPI staining shows the location of the nucleus. (JPG) [file pone.0026674.s007.jpg]
