# Supplementary material for: Suppressor of Cytokine Signalling-6 Promotes Neurite Outgrowth via JAK2/STAT5-Mediated Signalling Pathway, Involving Negative Feedback Inhibition
Source: PLoS One. 2011 Nov 17;6(11):e26674. doi: 10.1371/journal.pone.0026674 (PMC3219632; doi:10.1371/journal.pone.0026674)
Supplement: Figure S3 — Effect of SOCS6 on neuritic outgrowth of PC12 cells. (A) PC12 cells stable transfected with EGFP or SOCS6 EGFP were allowed to differentiate in the presence of NGF and observed under fluorescent microscope. (B, C) PC12 cells stably transfected with EGFP or with SOCS6-EGFP were differentiated with NGF in the presence and absence of IGF-1. Average length of primary, secondary and tertiary branches in EGFP and SOCS6 stably transfected PC12 cells and average number of neurites per cell. Neurite length was measured in randomly chosen cells. Statistical significance of the difference was determined using ANOVA. The result shows the mean ±SE of n = 3 combined experiments (**p<0.01, *p<0.05).Neurite lengths were measured by tracing individual neurites (as described in experimental procedures) and results are expressed as (B) average of total primary, secondary and tertiary neurite lengths or (C) average number of neurites per cell. Scale bar 5 µM. (PPT) [file pone.0026674.s003.ppt]

## Slide 1
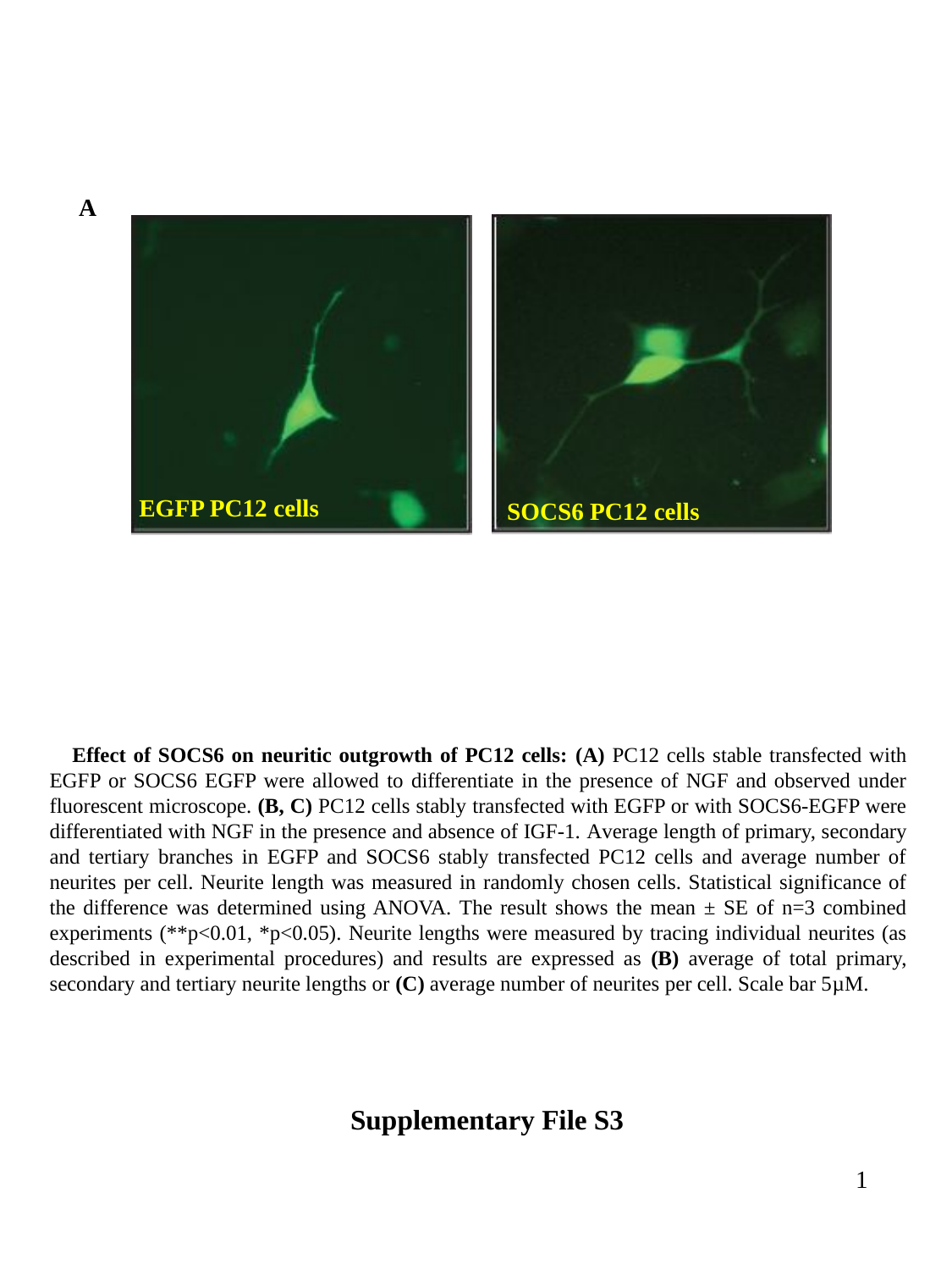

A
EGFP PC12 cells
SOCS6 PC12 cells
 Effect of SOCS6 on neuritic outgrowth of PC12 cells: (A) PC12 cells stable transfected with EGFP or SOCS6 EGFP were allowed to differentiate in the presence of NGF and observed under fluorescent microscope. (B, C) PC12 cells stably transfected with EGFP or with SOCS6-EGFP were differentiated with NGF in the presence and absence of IGF-1. Average length of primary, secondary and tertiary branches in EGFP and SOCS6 stably transfected PC12 cells and average number of neurites per cell. Neurite length was measured in randomly chosen cells. Statistical significance of the difference was determined using ANOVA. The result shows the mean ± SE of n=3 combined experiments (**p<0.01, *p<0.05). Neurite lengths were measured by tracing individual neurites (as described in experimental procedures) and results are expressed as (B) average of total primary, secondary and tertiary neurite lengths or (C) average number of neurites per cell. Scale bar 5µM.
Supplementary File S3
<number>

## Slide 2
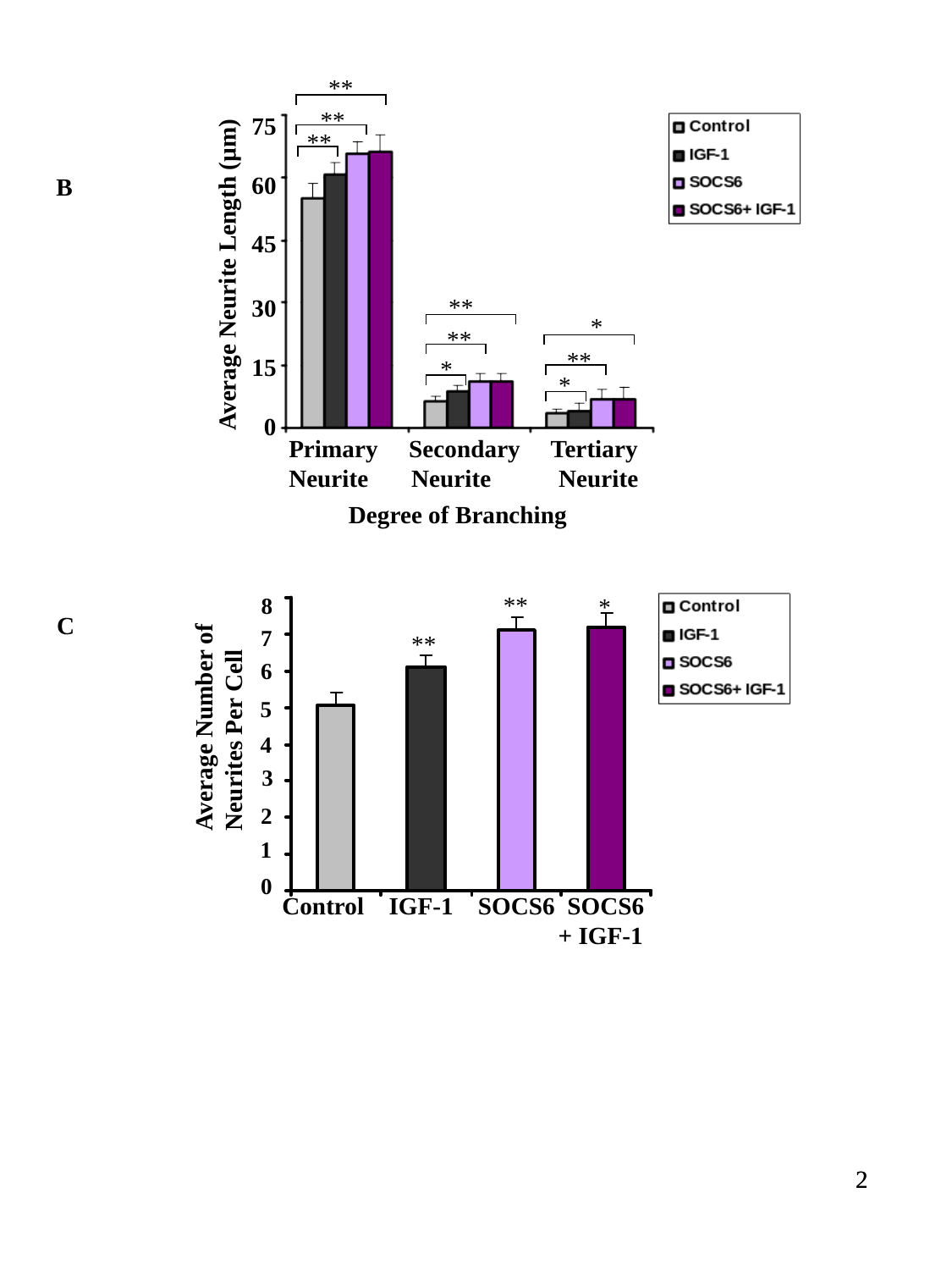

**
**
75
60
45
**
B
Average Neurite Length (µm)
**
30
15
 0
*
**
**
*
*
Primary Secondary Tertiary
Neurite Neurite Neurite
Degree of Branching
**
8
*
C
7
**
6
Average Number of
Neurites Per Cell
5
4
3
2
1
0
Control IGF-1 SOCS6 SOCS6
 + IGF-1
<number>
<number>
