# Supplementary material for: Suppressor of Cytokine Signalling-6 Promotes Neurite Outgrowth via JAK2/STAT5-Mediated Signalling Pathway, Involving Negative Feedback Inhibition
Source: PLoS One. 2011 Nov 17;6(11):e26674. doi: 10.1371/journal.pone.0026674 (PMC3219632; doi:10.1371/journal.pone.0026674)
Supplement: Figure S5 — Effect of AG490 on neuritic outgrowth of PC12 cells. PC12 cells were allowed to differentiate in the presence of 50 µMAG490 (Jak2/Stat3 inhibitor) for 4 days. (A) The cells were seen under light microscope. (B) Number of cells with neurites per field was counted. An average of 13 fields was taken. The untreated control was taken as 100%. (C and D) Neurite length was measured in randomly chosen cells. Statistical significance of the difference was determined using ANOVA. The result shows the mean ±SE of n = 3 combined experiments (**p<0.01, *p<0.05). Neurite lengths were measured by tracing individual neurites (as described in experimental procedures) and results are expressed as (C) average of total primary and secondary neurite lengths or (D) average number of neurites per cell. Scale bar, 5 µM. (E) PC12 cells were allowed to grow in the presence of increasing the concentration of AG490 (0 µM-100 µM) and MTT assay was performed. (PPT) [file pone.0026674.s005.ppt]

## Slide 1
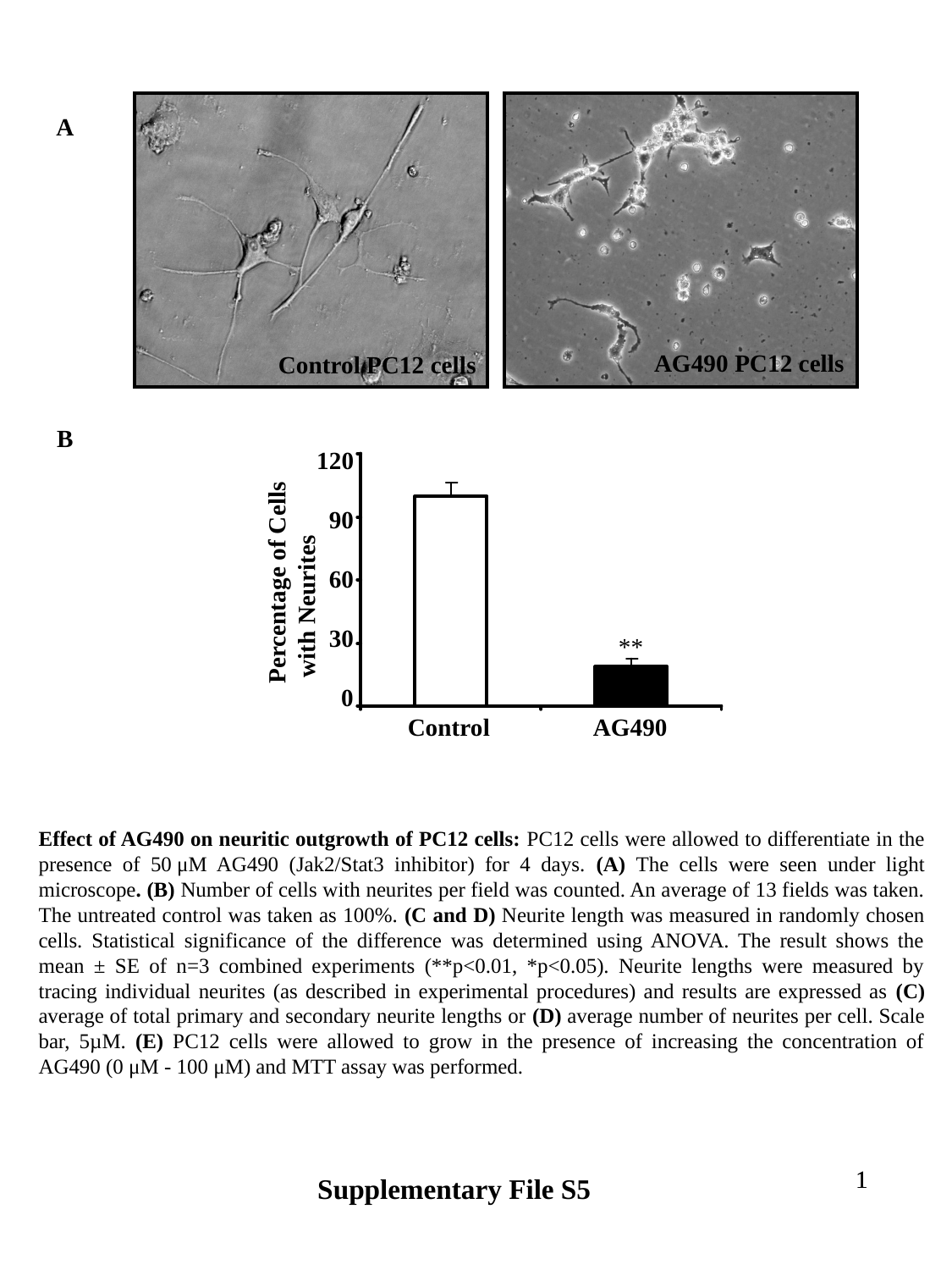

AG490 PC12 cells
Control PC12 cells
A
B
120
 90
 60
 30
 0
Percentage of Cells
 with Neurites
**
Control AG490
Effect of AG490 on neuritic outgrowth of PC12 cells: PC12 cells were allowed to differentiate in the presence of 50 μM AG490 (Jak2/Stat3 inhibitor) for 4 days. (A) The cells were seen under light microscope. (B) Number of cells with neurites per field was counted. An average of 13 fields was taken. The untreated control was taken as 100%. (C and D) Neurite length was measured in randomly chosen cells. Statistical significance of the difference was determined using ANOVA. The result shows the mean ± SE of n=3 combined experiments (**p<0.01, *p<0.05). Neurite lengths were measured by tracing individual neurites (as described in experimental procedures) and results are expressed as (C) average of total primary and secondary neurite lengths or (D) average number of neurites per cell. Scale bar, 5µM. (E) PC12 cells were allowed to grow in the presence of increasing the concentration of AG490 (0 μM - 100 μM) and MTT assay was performed.
<number>
<number>
Supplementary File S5

## Slide 2
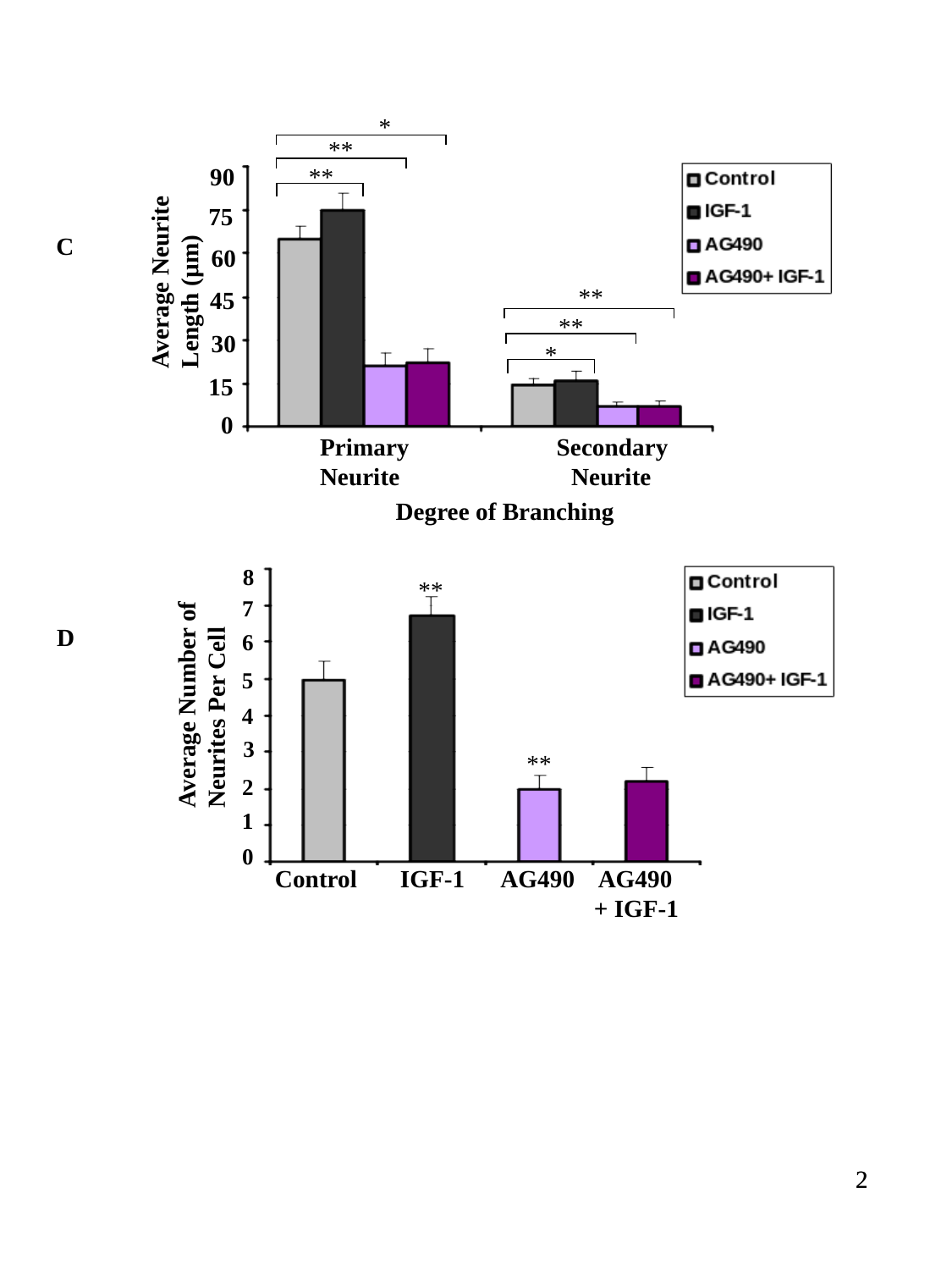

*
**
90
**
75
C
60
Average Neurite
Length (µm)
**
45
**
30
*
15
0
Primary Secondary
Neurite Neurite
Degree of Branching
8
**
7
D
6
5
Average Number of
Neurites Per Cell
4
3
**
2
1
0
Control IGF-1 AG490 AG490
 + IGF-1
<number>
<number>

## Slide 3
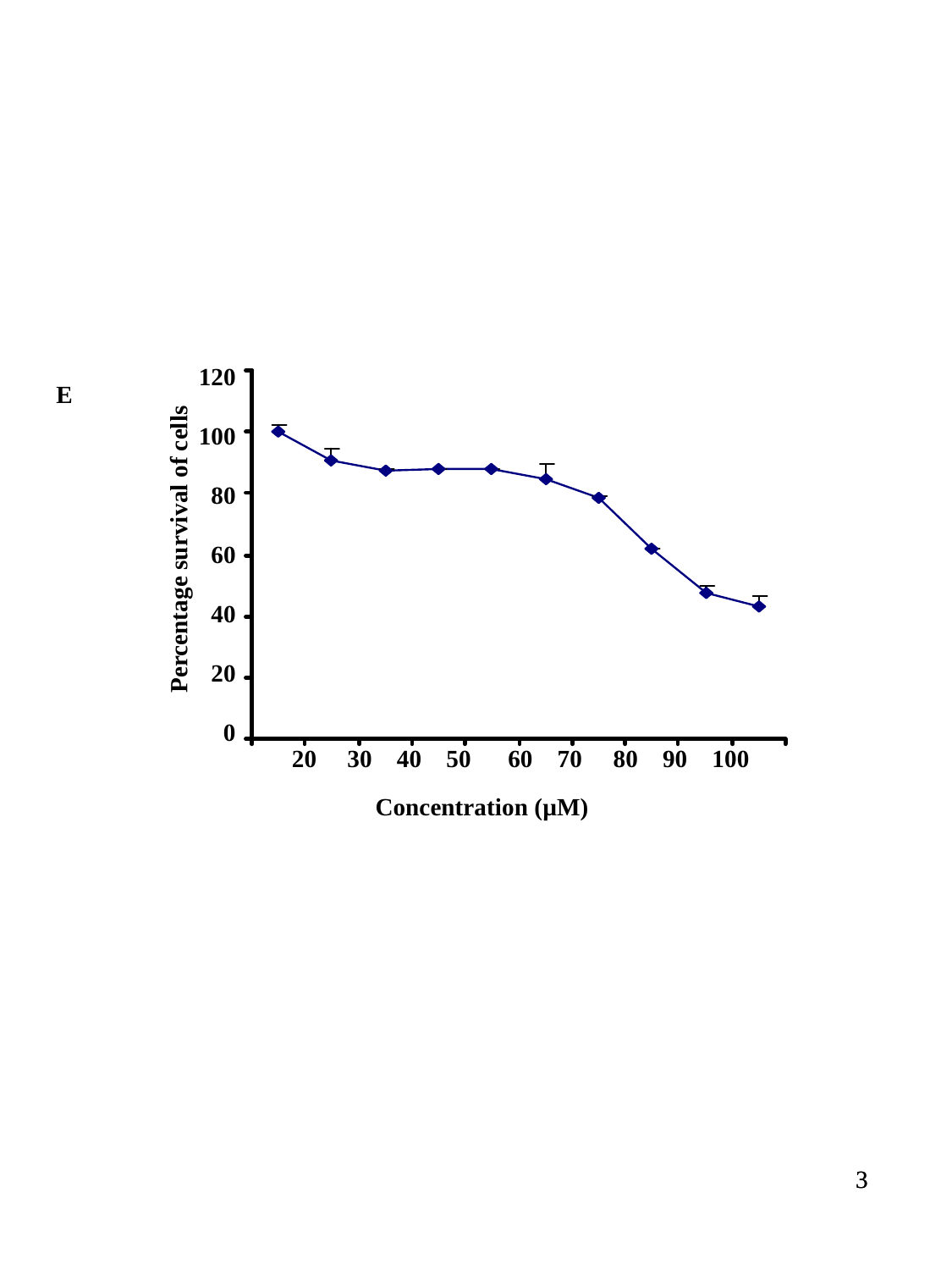

120
100
 80
 60
 40
 20
 0
E
Percentage survival of cells
20 30 40 50 60 70 80 90 100
Concentration (µM)
<number>
<number>
